# Weight variability and cardiovascular outcomes: a systematic review and meta-analysis

**DOI:** 10.1186/s12933-022-01735-x

**Published:** 2023-01-09

**Authors:** Robert J. Massey, Moneeza K. Siddiqui, Ewan R. Pearson, Adem Y. Dawed

**Affiliations:** Population Health & Genomics, School of Medicine, University of Dundee, Dundee, DD1 9SY UK

**Keywords:** Weight variability, Obesity, Cardiovascular disease, Type II diabetes, Meta-analysis

## Abstract

The association between body weight variability and the risk of cardiovascular disease (CVD) has been investigated previously with mixed findings. However, there has been no extensive study which systematically evaluates the current evidence. Furthermore, the impact of ethnicity and type 2 diabetes on this phenomena has not yet been investigated. Therefore, the aim of this study was to comprehensively evaluate the effect of weight variability on risk of CVD (any cardiovascular (CV) event, composite CV outcome, CV death, Stroke, Myocardial Infarction) and the influence of ethnicity and type 2 diabetes status on the observed association. A systematic review and meta-analysis was performed according to the meta-analyses of observational studies in epidemiology (MOOSE) guidelines. The electronic databases PubMed, Web of Science, and the Cochrane Library were searched for studies that investigated the relationship between body weight or BMI variability and CV diseases using Medical Subject Headings (MeSH) terms and keywords. The relative risks (RRs) for the outcomes were collected from studies, pooled, and analysed using a random-effects model to estimate the overall relative risk. Of 5645 articles screened, 23 studies with a total population of 15,382,537 fulfilled the prespecified criteria and were included. Individuals in the highest strata of body weight variability were found to have significantly increased risk of any CV event (RR = 1.27; 95% Confidence Interval (CI) 1.17–1.38; P < 0.0001; I^2^ = 97.28%), cardiovascular death (RR = 1.29; 95% CI 1.03–1.60; P < 0.0001; I^2^ = 55.16%), myocardial infarction (RR = 1.32; 95% CI 1.09–1.59; P = 0.0037; I^2^ = 97.14%), stroke (RR = 1.21; 95% CI 1.19–1.24; P < 0.0001; I^2^ = 0.06%), and compound CVD outcomes (RR = 1.36; 95% CI 1.08–1.73; P = 0.01; I^2^ = 92.41%). Similar RRs were observed regarding BMI variability and per unit standard deviation (SD) increase in body weight variability. Comparable effects were seen in people with and without diabetes, in White Europeans and Asians. In conclusion, body weight variability is associated with increased risk of CV diseases regardless of ethnicity or diabetes status. Future research is needed to prove a causative link between weight variability and CVD risk, as appropriate interventions to maintain stable weight could positively influence CVD.

**Supplementary Information:**

The online version contains supplementary material available at 10.1186/s12933-022-01735-x.

## Introduction

Obesity is the strongest risk factor for both T2D and CVD [1]. Adolescents with a BMI over 30 at 18 years of age have a greater than 50% risk of developing T2D during their life [1]. Furthermore, the lifetime risk of incident CVD has been shown to be higher in overweight and obese adults, with hazard ratios of 1.67 (95% CI 1.55–1.79) and 1.85 (95% CI 1.72–1.99) for men and women with obesity, respectively [2]. As T2D independently increases the risk of CVD [3], the combination of obesity and T2D can be considered to have a particularly adverse effect on an individual’s cardiovascular health.

Due to the large contribution that obesity has to both T2D and CVD, weight loss is commonly recommended as a lifestyle intervention. However, weight loss is frequently followed by weight gain leading to patterns of weight cycling. Whether variability in body weight is associated with worse CV prognosis is controversial. While there are multiple studies that reported an association between weight variability and CVD [4, 5], others failed to corroborate these findings [6–8]. Currently, there has been no extensive study which systematically evaluates available evidence. In addition, the impact of ethnicity and T2D has not been investigated yet. Therefore, the aim of this study is to comprehensively evaluate the effect of weight variability on risk of CVD and, the influence of ethnicity and T2D status on any observed association.

## Methods

Before data collection was instigated, the purpose and preliminary protocol of this meta-analysis was registered with the PROSPERO International prospective register of systematic reviews (registration number CRD42021284787). This systematic review and meta-analysis assessing the association between body weight variability and cardiovascular (CV) events was conducted according to the Meta-analysis of Observational Studies in Epidemiology (MOOSE) Guidelines [9].

### Search strategy and study selection

In this systematic review and meta-analysis, we searched PubMed, Web of Science, and the Cochrane Library for studies that investigate association between weight/BMI variability and CV disease up to 26th of October 2021. For key terms and the search methods used see Additional file 1: Appendix S1. A list of the inclusion and exclusion criteria can be found in Additional file 1: Appendix S3. Studies that fulfilled the inclusion criteria were included. If full studies were not available electronically online, the primary authors were contacted via email to request a copy. For a description of the emails, see Additional file 1: Appendix S2. No response was received from contacted authors, however from the available abstracts we do not believe these exclusions have introduced bias into our results.

Our analysis included any study that investigated the association between weight variability and the risk of subsequent cardiovascular events in individuals aged  ≥ 18 years. Studies must have had at least 500 participants and a minimum follow-up period of 1 year. Included studies must have published relative risk (RR) estimates such as risk ratios, rate ratios, odds ratios, or hazard ratios with associated 95% confidence intervals (CIs) for events. Any definition and measurement of weight variability (i.e. variability independent of the mean (VIM), coefficient of variation (CoV), average successive variability (ASV), standard deviation (SD), and root mean squared error of residual variation (RMSE)) was considered. Weight variability was recorded either as a continuous or categorical variable. The effects of weight variability were reported as either the estimated RR of cardiovascular event per unit increase in SD of variability or by splitting the study population into strata of variability and then using the least variable or stable group as a reference. For descriptions of the definitions and measurements of weight variability used by the studies included in this analysis, see Additional file 1: Table S1. The primary outcome of our study was the development of any new singular (i.e. non-composite) cardiovascular event. Secondary outcomes were cardiovascular death, MI, stroke, and the most composite CVD outcomes recorded by studies. Studies not published in English were not included.

### Data extraction

From the studies that met all eligibility criteria, data concerning the name of the primary author, the year of publication, total and strata sample sizes, description of study population, whether the study investigated BMI or weight variability, definition and calculation of variability, definition of CV outcome, number of events, the RRs of the most adjusted model and associated 95% CIs, and the covariates included in the model were extracted.

Outcomes recorded were categorised based on how they were defined in their original studies. Any recorded MI, cardiovascular death, or stroke events that were defined as such by their original papers were grouped by these definitions in the secondary analyses. This method had three notable exceptions, where one report of ischaemic heart disease deaths [10], one report of cerebrovascular deaths [10], and two reports of coronary heart disease deaths [11], have been included in the cardiovascular death analysis. Similarly, two separate studies recorded RRs for CVD, and these reports have been treated as a compound outcomes and thus included in the most composite CVD outcome analysis [6, 12].

### Statistical methods

Summary RR statistics from the individual studies included were pooled based on whether the study they were taken from had investigated variability in body weight or BMI. The standard error (SE) of each collected RR was calculated from respective 95% CIs using the formula $SE= \frac{(upper\,CI\,limit - lower\,CI\,limit)}{3.92}$. These estimates were combined using random effects model separately for weight and BMI variability. First, we compared the RR of CV outcomes for the most variable group versus the least variable group [4–8, 10–25]. Then RRs per + 1 SD increase of the unit of weight variability [4, 5, 14, 19, 26, 27]. This second analysis was not performed for BMI variability as there are no included studies that report recording CV risks per + 1 SD of BMI variability. Lissner et al*.* produced estimated RRs that were only available stratified by sex, and as such the male and female RRs were treated as separate reports [11]. Nam et al*.* took BMI and body weight measurements for all participants and treated these 2 sets of data as 2 separate reports, and we have used these reports separately in the respective BMI/body weight variability analyses [20]. When a study recorded RR estimates for 2 or more separate single CVD events (e.g. MI, Stroke, etc.), these were also treated as separate reports. When a study reported multiple compound outcomes, only the most composite outcome (i.e. the composite outcome containing the greatest number of different cardiovascular outcomes) was collected. For each report gathered, only the RR estimates from the most adjusted models available were included in the final analyses. Meta-analysis was performed using the ‘metafor’ package in R (version 4.1.1) [28].

Weight cycling was defined and measured in studies via 2 different measures: categorical and continuous. Continuous definitions included CoV [11, 13, 20], ASV [4, 5, 12, 14, 25, 27], RMSE [10, 26], SD [15], and VIM [16–19, 21]. Categorical definitions included a BMI loss of  ≥ 2 followed by a gain of  ≥ 2 or vice-versa [6], a weight loss of at least 10 lbs (4.5 kg) at least 3 times [22], a BMI loss of  ≥ 4% from baseline to midpoint follow-up and then a BMI gain of  ≥ 4% from midpoint follow-up to last visit or vice-versa [7], a sum of deviations  > 5.04 for those with  < 3.0 unit difference from their initial to final BMI [23], being in the top quintile of VIM-defined body-weight variability [8], and experiencing a loss then a gain in body weight or vice versa [24].

### Quality assessment

The Newcastle–Ottawa Quality Assessment Scale for Cohort Studies (NOS) was used for assessing the quality and bias of the studies included in the final analyses [29]. For the results of this analysis, see Additional file 1: Table S3. Heterogeneity between studies was estimated for the primary and secondary outcome analyses using the I^2^ statistic, Egger’s regression test, and funnel plot. If heterogeneity was observed, the Duval and Tweedie trim-and-fill method was used to adjust for publication bias [30].

### Sensitivity analysis

Sensitivity analyses was performed to investigate the effect of ethnicity, diabetes status, metric of variability (e.g. CoV, ASV, etc.), quantile of variability, adjustment for each participant’s average BMI or change in BMI, and pre-existing cardiovascular risk or disease. A univariate meta-regression was also performed to analyse the effect of the average BMI of the included studies on the relative risk observed. These analyses were only performed on summary RRs collected from studies that investigated body weight variability due to lack of data regarding BMI variability). Sensitivity analysis by ethnicity was done in White Europeans and Asians as there are not enough studies for the analysis of any other ethnic groups.

## Results

### Study selection

**Fig. 1:**
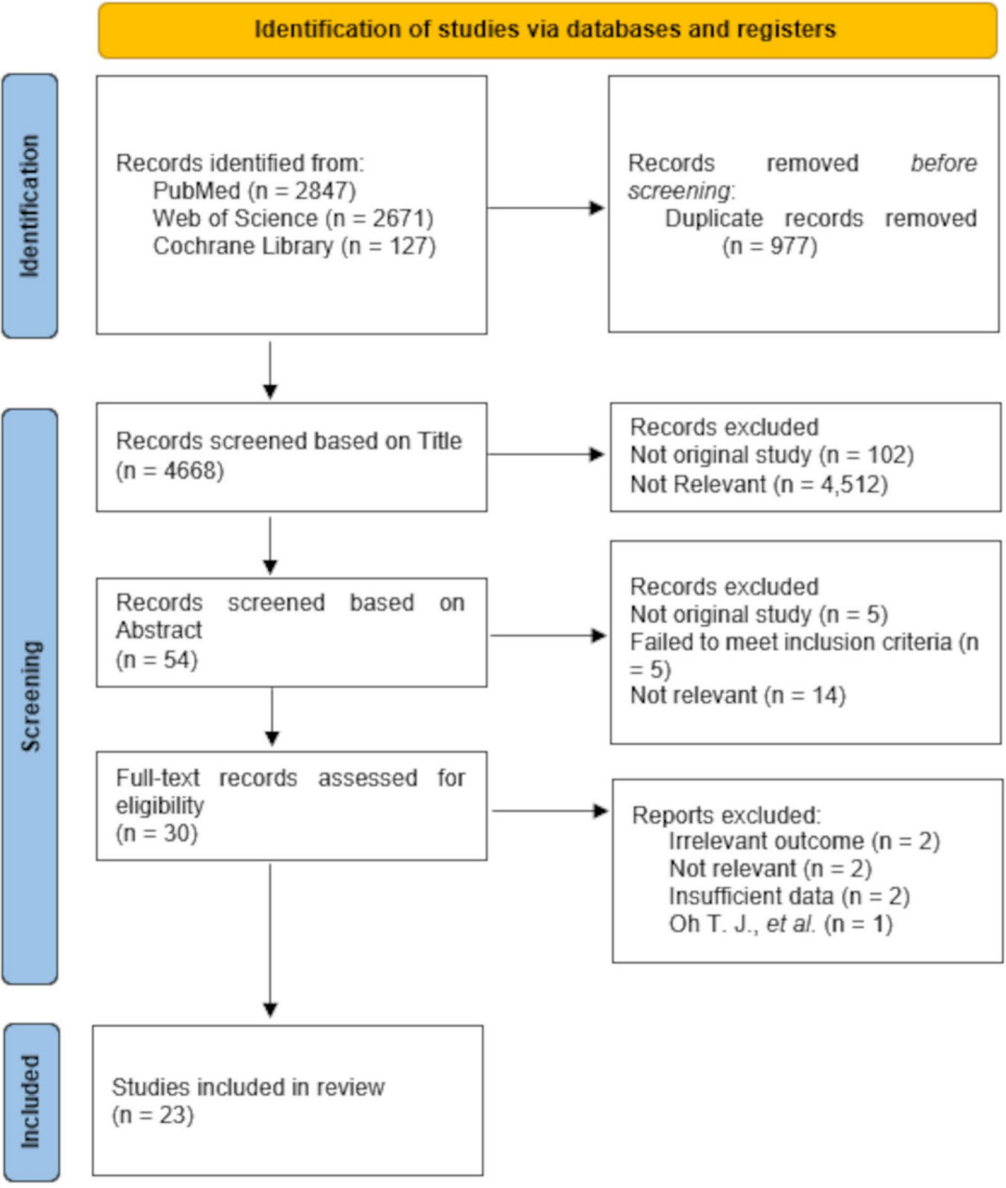
A flow chart of the study selection process

Of the 5645 articles screened for eligibility, 23 studies with 15,382,537 individuals were included in the final meta-analyses [4–8, 10–27] (Fig. 1). From these 23 studies, 21 studies were for singular cardiovascular events [4–8, 10–21, 23, 24, 26, 27], 11 for cardiovascular death [4, 6, 7, 10–12, 14, 20, 23, 24, 26], 8 for MI events [4, 5, 13–17, 21], 7 for any stroke event [5, 6, 14–17, 21], and 10 for composite CVD outcomes [4–6, 8, 11, 12, 14, 22, 25, 27]. Of these 23 studies, 17 investigated body weight variability [4–6, 11, 13–22, 25–27], whilst 7 investigated BMI variability [7, 8, 10, 12, 20, 23, 24]. The study performed by Nam et al*.* [20] contributed data towards both body weight and BMI variability analyses [20]. Despite meeting inclusion criteria, estimated RRs from the 2019 study by Oh et al. were not included in the final analyses due to only recording estimated RRs per + 1 increase in average successive variability (ASV), and as such not compatible with our analysis [31].

### Study characteristics

The recorded average (mean or median) age of the participants within studies ranged from 35 to 72 years. The average weight of participants ranged from 63.5 kg to 92.5 kg. The average BMI of participants ranged from 22.1 to 33.2 kg/m^2^. Almost every study included in this analysis had > 50% male participation, except for 3 [8, 11, 22]. Of the included studies, 12 had > 50% White participants [4, 5, 8, 11, 13–15, 22–24, 26, 27], and 11 had > 50% East Asian participants [6, 7, 10, 12, 16–21, 25]. The participants of 6 of the studies had been previously diagnosed with T2D [13–15, 18, 21, 27], whilst 1 more provided separate statistics for the participants within the study who had been previously diagnosed with T2D [12]. The average follow up time for the included studies ranged from 3.7 to 32 years (See Additional file 1: Table S2). Of the 23 studies, 16 were judged as having a high quality (≥ 7) based on the Newcastle–Ottawa scale (See Additional file 1: Table S4). Sponholtz et al*.* separated their participant population based upon whether they were obese, and as such generated 2 reports per cardiovascular outcome. Similarly, Wannamethee et al*.* stratified their population based upon whether their participants initially lost or gained weight [24], Lissner et al*.* stratified their population by gender [11], and Youk et al*.* stratified by diabetes status [12], and as such each of these studies produced 2 reports per cardiovascular outcome. In total, 58 reports regarding cardiovascular outcomes were collected from the 23 studies. Of these 58 reports, 47 reported on a singular (i.e. non-compound) recorded CV event, and 11 reported composite CVD outcomes. Of the 47 reports of singular CV events, 17 reports were for CV death, 8 for MI, and 7 for stroke.

### Body weight and BMI variability were associated with increased risk of any cardiovascular event

**Fig. 2:**
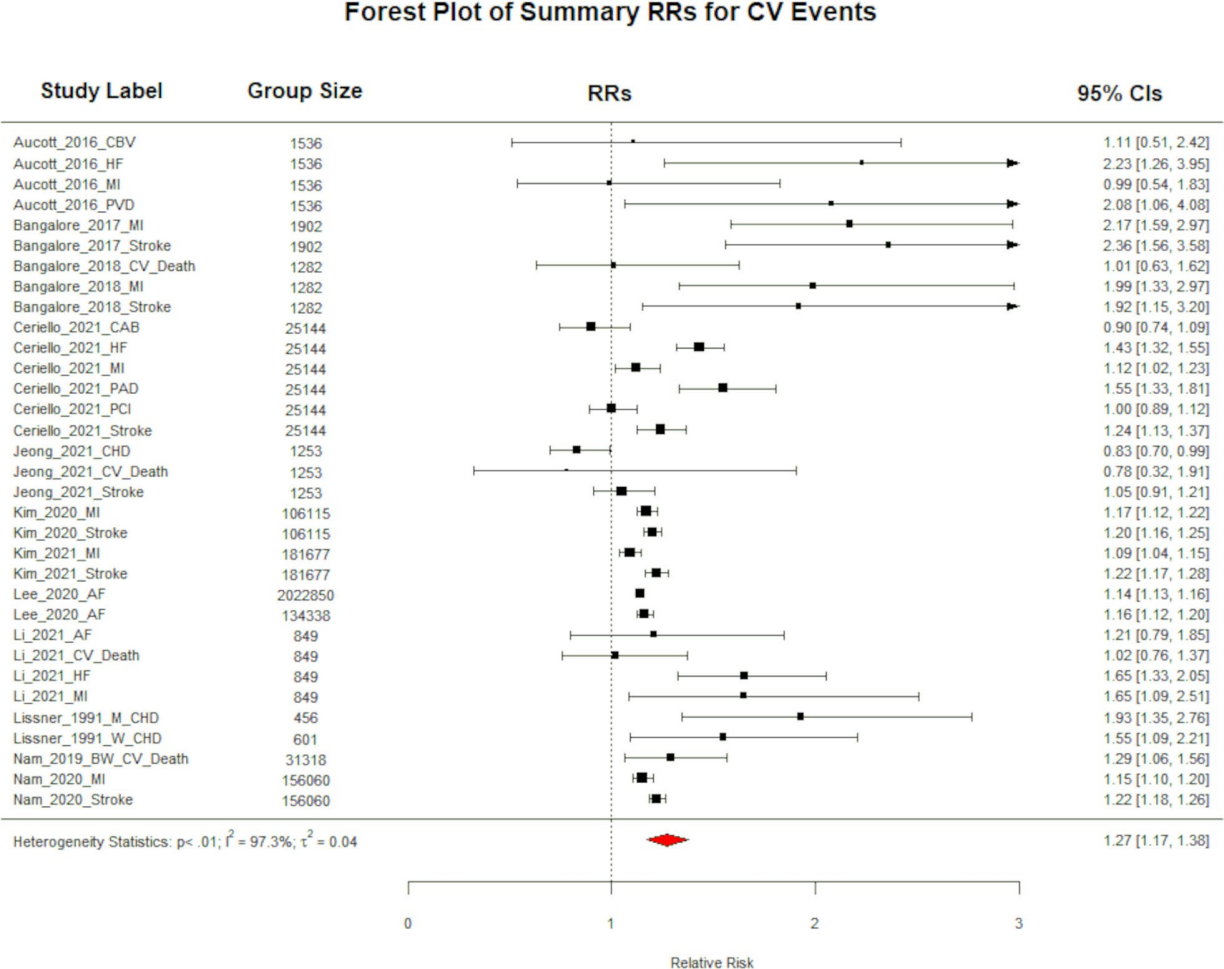
A forest plot showing the summative risk of any cardiovascular event associated with being in the most variable body weight group compared to the least variable. The number of participants in the most variable group are shown in the column “Group Size”. RR = 1.27; 95% CI 1.17–1.38; P < 0.0001; Significant Heterogeneity (I.^2^ = 97.28%; P < 0.0001)

A total of 21 studies (15 for body weight and 6 for BMI) consisting of 15,141,102 individuals investigated the association between weight/BMI variability and any CV outcomes. Compared to the least variable group, the summary RR for any CV outcomes for people in the most variable group of body weight was 1.27 (95% CI 1.17–1.38; P < 0.0001; I^2^ = 97.28%; P < 0.0001 for heterogeneity; Fig. 2). A similar summary RR of 1.39 (95% CI 1.17–1.64; P < 0.0001; I^2^ = 76.39%; P < 0.0001 for heterogeneity; See Additional file 1: Figure S2a) was found when the variability was defined using BMI. The summary RR estimate for the association between per + 1 SD increase in unit of body weight variability and any cardiovascular event was 1.16 (95% CI 1.06–1.26; P < 0.0001; I^2^ = 94.70%; P = 0.0013 for heterogeneity; See Additional file 1: Figure S1a).

### Body weight and BMI variability were associated with increased risk of cardiovascular death

**Fig. 3:**
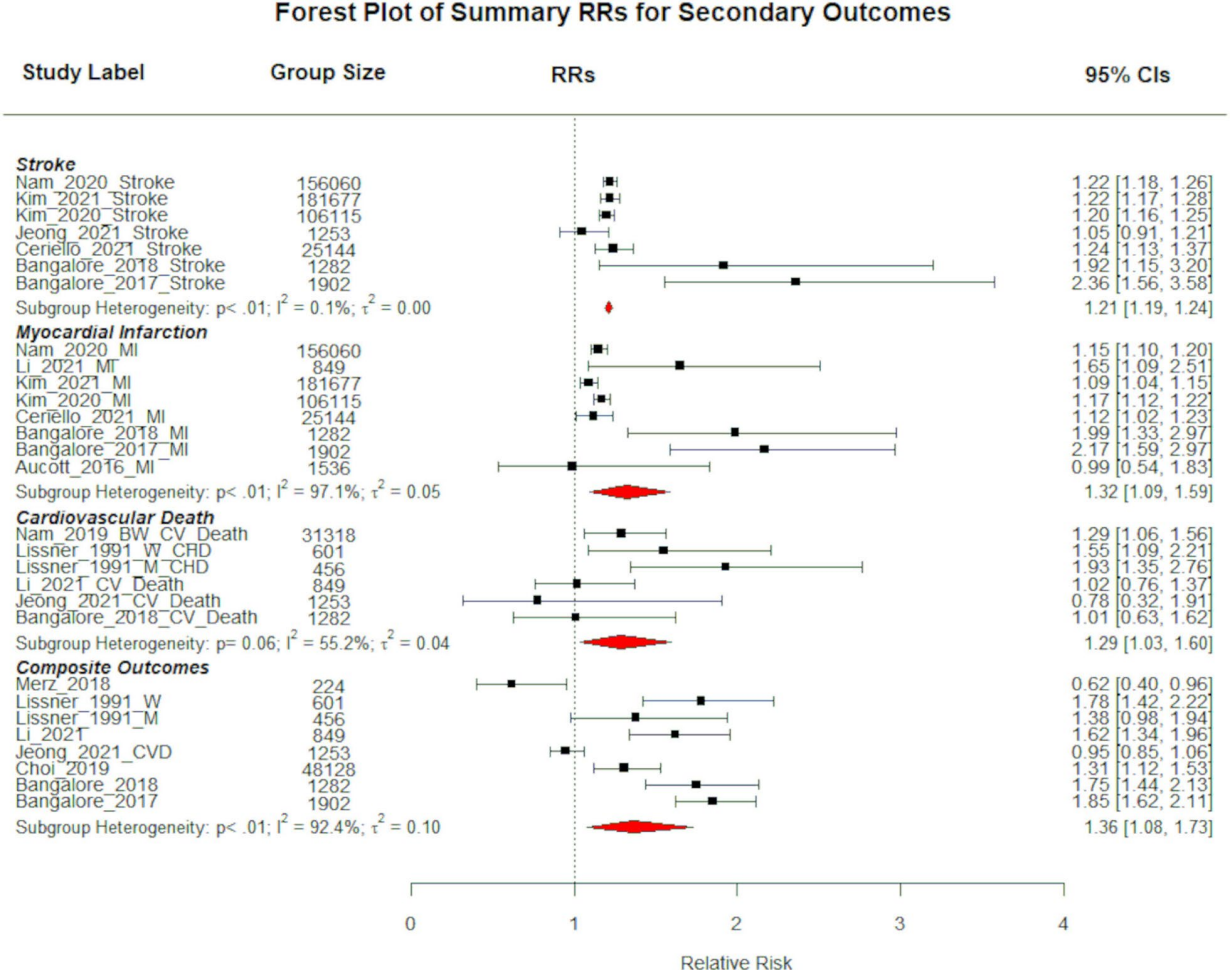
A compound forest plot showing the summative risk of the secondary outcomes associated with being in the most variable body weight group compared to the least variable. The subheadings “Stroke”,”Myocardial Infarction”, “Cardiovascular Death”, and “Composite Outcomes” are followed by the reports included in the respective sub-analysis. The number of participants in the most variable group are shown in the column “Group Size”. CV Death RR = 1.29 (95% CI 1.03–1.60; P = 0.0233; I^2^ = 55.16%; P for heterogeneity = 0.062). MI RR = 1.32 (95% CI 1.09–1.59; P = 0.0037; I^2^ = 97.14%; P for heterogeneity < 0.0001). Stroke RR = 1.21 (95% CI 1.19–1.24; P < 0.0001; I^2^ = 0.06%; P for heterogeneity = 0.0073). Most composite CV outcome RR = 1.36 (95% CI 1.08–1.73; P = 0.01; I.^2^ = 92.41%; P for heterogeneity < 0.0001)

A total of 11 studies (5 for body weight variability and 6 for BMI variability) consisting of 633,592 participants investigated the association between weight/BMI variability and risk of CV death. Compared to the least variable group, the summary RR for CV deaths for the most variable group of body weight and BMI was 1.29 (95% CI 1.03–1.60; P < 0.0001; I^2^ = 55.16%; P = 0.062 for heterogeneity; Fig. 3) and 1.27 (95% CI 1.09–1.49; P = 0.0027; I^2^ = 68.51%; P = 0.002 for heterogeneity; See Additional file 1: Figure S2b), respectively. The summary RR for CV Deaths per + 1 SD increase in body weight variability was 1.11 (95% CI 1.02–1.21; P = 0.0132; I^2^ = 49.66%; P for heterogeneity = 0.1359; See Additional file 1: Figure S1b).

### Body weight variability was associated with increased risk of myocardial infarction

There were eight studies with a total population of 5,742,933 that investigated the association between body weight variability and MI. The summary RR for MI associated with being in the most variable strata of body weight compared to the least variable strata was 1.32 (95% CI 1.09–1.59; P = 0.0037; I^2^ = 97.14%; P for heterogeneity < 0.0001; Fig. 3), and the summary RR per + 1 SD increase in body weight variability was 1.14 (95% CI 0.92–1.42; P = 0.2234; I^2^ = 82.32%; P for heterogeneity = 0.0174; Additional file 1: Figure S1b). No study that investigated BMI variability reported RR for MI.

### Body weight variability was associated with increased risk of stroke

There were 7 studies consisting of 5,779,027 subjects that investigated the association between body weight variability and risk of stroke. The summary RR for stroke associated with being in the most variable strata of body weight compared to the least variable strata was 1.21 (95% CI 1.19–1.24; P < 0.0001; Fig. 3). Significant heterogeneity was detected in this analysis (I^2^ = 0.06%; P for heterogeneity = 0.0073). No study that investigated BMI variability recorded RR estimates for stroke outcomes. Similarly, it was not possible to perform a meta-analysis on the risk of stroke per + 1 SD increase in body weight variability, as there is only 1 study that reported RR for stroke [5].

### Body weight variability and risk of composite cardiovascular outcomes

Eight studies consisting of 339,566 participants reported association between body weight variability and composite CV outcomes. The RR for composite CVD outcomes associated with being in the most variable body weight group compared to the least variable was 1.36 (95% CI 1.08–1.73; P = 0.01; I^2^ = 92.41%; P for heterogeneity < 0.0001; Fig. 3), and the RR of composite CVD outcomes associated per + 1 SD increase in body weight variability was 1.14 (95% CI 1.04–1.25; P = 0.0047; I^2^ = 91.77%; P for heterogeneity < 0.0001; See Additional file 1: Figure S1b). There was only one study that investigated the association between BMI variability and composite CV outcome [8] and thus a meta-analysis is not performed.

### The association between weight variability and CV outcomes was not modified by ethnicity or diabetes status

Given ethnicity and diabetes status are known risk factors for CV outcomes [32, 33], we performed subgroup analyses stratified by ethnicity and diabetes status. Due to a lack of data on other ethnicities, this analysis was performed in White Europeans and East Asians. We observed that both ethnicity and diabetic status generally had no significant effect on the observed association between body weight variability and CV events (See Additional file 1: Figures S3 and S4). Compared to the group with the least variability, a significantly higher risk of any CV event was observed in the group with the highest degree of body weight variability in both the White (RR = 1.42; 95% CI 1.25–1.62; P < 0.0001; See Additional file 1: Figure S3a) and East Asian populations (RR = 1.16; 95% CI 1.12–1.19; P < 0.0001;

See Additional file 1: Figure S3b). The RR for CV death was not statistically significant in either the Whites (RR = 1.33; 95% CI 0.97–1.83; P = 0.0741; See Additional file 1: Figure S3c) or the East Asians (RR = 1.22; 95% CI 0.90–1.66; P = 0.2022; See Additional file 1: Figure S3d). The RR of composite CVD outcome in East Asians was also not significant (RR = 1.11; 95% CI 0.81–1.52; P = 0.5154; See Additional file 1: Figure S3d). This is most likely due to a lack of power after stratification.

Similar results were obtained after stratifying by diabetes status. The RR for any CV event in the most variable group compared to the least variable group with diabetes was 1.25 (95% CI 1.13–1.38; P < 0.0001; I^2^ = 98.03%; P for heterogeneity < 0.0001) and in non-diabetics it was 1.29 (95% CI 1.14–1.46; P < 0.0001; I^2^ = 98.03%; P for heterogeneity < 0.0001). No differential association by diabetes status was also observed between weight variability and MI, however the association between weight variability and stroke was observed to be insignificant in the non-diabetic population (RR = 1.31; 95% CI 0.99–1.72; P = 0.0566; I^2^ = 98.35%; P for heterogeneity = 0.0029) (See Additional file 1: Figure S4c and d). Only one of the papers included in this analysis investigated cardiovascular death or composite cardiovascular outcomes in individuals with type II diabetes [14], and as such these outcomes were not suitable for subgroup meta-analysis. Therefore, the effect of diabetes status on the risk of these outcomes cannot be analysed.

### Sensitivity analyses

Given different studies capture weight variability using different metrics (i.e. ASV, SD, CoV, VIM, RMSE), we performed sensitivity analysis after stratification by exposure definition. Overall, exposure definition had little impact on the results, with a few notable exceptions (See Additional file 1: Figure S5a–i). When ASV was used, the summary RRs associated with the most variable strata of body weight (compared to the least variable) for both MI and stroke were significantly higher, with the RR for MI 1.97 (95% CI 1.60–2.44; P < 0.0001; See Additional file 1: Figure S5b) and stroke 2.17 (95% CI 1.57–3.00; P < 0.0001; See Additional file 1: Figure S5b). However, when ASV was used as a measure of variability in the per + 1 SD increase in body weight variability analysis, the summary RR for MI became insignificant (See Additional file 1: Figure S5i). Similarly, when ASV was used as a measure of variability, the summary RR for CV death became insignificant (See Additional file 1: Figure S5b and i). These changes are most likely explained by a lack of power in these sub-analyses. Of note however is the general trend of decreased heterogeneity of results observed after stratifying by metric of effect, which suggests that this difference in study methodology is a large contributor to the difference in result we observe between studies.

An important question to address when interpreting these results is how much the specific quantile used in the most variable group versus the least variable group affects the observed association. To explore this, we stratified the most variable versus least variable body weight analysis based on the quantile used by the included studies. Of the 15 included studies that compared quantiles, 6 compared quintiles, 7 compared quartiles, 1 compared tertiles, and 1 compared medians (See Additional file 1: Table S3). We therefore stratified the analysis by studies that investigated quintiles or quartiles. Due to a lack of included studies investigating BMI variability, this sensitivity analysis was only performed on studies that investigated body weight variability. We also removed studies that did not compare the highest quantile to the lowest quantile, and instead compared the top quantile against the combined lower quantiles [18, 20]. This results in 3 studies being included in the quintile analysis [5, 14, 25], and 5 included in the quartile analysis [15–17, 19, 21]. Compared to the quintile with the least variability, a significantly higher risk of any CV event was observed in the quintile with the highest degree of body weight variability (RR = 1.86; 95% CI 1.41–2.44; P < 0.001; I^2^ = 54.40%; P for heterogeneity = 0.0725). A similar result was found in studies that compared quartiles (RR = 1.18; 95% CI 1.11–1.25; P < 0.0001; I^2^ = 95.30%; P for heterogeneity < 0.0001) (Additional file 1: Figure S6a and b). When comparing the risks of secondary outcomes, all outcomes remained significant in both the quintile and quartile strata, however a general trend was found where the risk observed in the quintile group was higher than that of the same outcome in quartile group. While this difference in RR between studies that compared quintiles and quartiles could reflect the true impact of weight variability on CV outcomes in the top 20% of the population, it could also be due to differences in sample sizes or unseen bias as studies that compared quartiles are largely from similar studies. Therefore, further investigation in a well powered prospective study is warranted (Additional file 1: Figure S6c and d).

One of the key questions when investigating weight variability is to what degree is the increased risk of CVD observed due to a general increase or decrease in body weight rather than a variability in weight. To explore this question, we stratified our analysis into studies that adjusted for average BMI or change in BMI versus studies that did not control for these covariates. When compared to the group with the least variability, a significantly higher risk of any CV event was observed in the group with the highest degree of body weight variability in studies that adjusted for BMI (RR = 1.60; 95% CI 1.37–1.87; P < 0.0001; I^2^ = 52.50%; P for heterogeneity = 0.0104; See Additional file 1: Figure S8a) and studies that did not (RR = 1.16; 95% CI 1.09–1.23; P < 0.0001; I^2^ = 95.63%; P for heterogeneity < 0.0001;

See Additional file 1: Figure S8c). When comparing secondary outcomes between these two strata, we found that the observed relative risk were similar, with a few exception (Additional file 1: Figure S8b and d). After stratification, the relative risk of CV death associated with weight variability was found to be insignificant in both groups, however this is most likely due to reduced power post-stratification. Similarly, the risk of composite cardiovascular outcomes was found to be insignificant in the unadjusted strata, again most likely due to reduced power post-stratification. An interesting observation is that the relative risks reported in the adjusted group were typically higher than those in the unadjusted group, however, this could be mostly explained by other differences between these two groups. For example, all of the studies in the adjusted analysis included participants of White Europeans and 80% of the participants in the unadjusted analysis had East Asian ancestry. In addition, the adjusted studies were generally smaller than the unadjusted studies, with a total adjusted population of 50,095 and an unadjusted population of 14,650,699.

Of the studies included in this analysis, several were performed on populations with pre-existing CVD. It is therefore important to investigate whether weight variability has a differential effect on risk of CVD among populations with high risk of CVD at baseline versus populations with no known CVD. Compared to the group with the least variability, a significantly higher risk of any CV event was observed in the group with the highest degree of body weight variability in both populations with pre-existing CVD (RR = 1.59; 95% CI 1.29–1.96; P < 0.0001; I^2^ = 65.75%; P for heterogeneity = 0.0035; See Additional file 1: Figure S7a) and no known CVD (1.19; 95% CI 1.11–1.26; P < 0.0001; I^2^ = 95.49%; P for heterogeneity < 0.0001; See Additional file 1: Figure S7c). The observed relative risk of secondary outcomes between these two groups were largely similar, with a few exceptions (See Additional file 1: Figure S7b and d). The relative risk of CV death associated with weight variability was found to be insignificant in the group with pre-existing CVD risk. The risk of composite cardiovascular outcomes associated with weight variability in both groups was found to be insignificant after stratification. However, these differences could be explained by a reduced power after stratification.

To further explore this question, we performed a univariate meta-regression to investigate whether the observed relative risk of CVD associated with weight variability was correlated on the average age of the study populations (See Additional file 1: Figure S9a–e). The relative risk of any CV event associated with weight variability was found to be significantly positively correlated with average age (β = 0.0081; P = 0.0345; R^2^ = 0.028). No significant correlation between average age and relative risk associated with weight variability was observed for any of the secondary outcomes.

### Heterogeneity and bias analysis

As heterogeneity was significant in the analysis of the RRs of the primary and secondary outcomes associated with being in the top strata of body weight variability, it was important to investigate whether this heterogeneity was due to publication bias. As such, Egger’s regression test and funnel plots were created for these analyses. Egger’s regression found no funnel plot asymmetry for the CV event (z = 1.7567; P = 0.079; See Additional file 1: Figure S10a), CV death (z =  −1.0027; P = 0.316; See Additional file 1: Figure S10b), MI (z = 1.1849; P = 0.236; See Additional file 1: Figure S10c), or the most composite CVD outcomes (z =  −1.7294; P = 0.0837; See Additional file 1: Figure S10e) analyses, however, significant asymmetry was found for the analysis of stroke (z = 2.9287; P = 0.0034; See Additional file 1: Figure S10d). As such, the Duval and Tweedie trim-and-fill method was employed in order to estimate the effect that hypothetical missing publications would have on the summary RR estimate. This analysis found no change in the estimated summary RR of any cardiovascular event associated with degree of body weight variability (RR = 1.21; 95% CI 1.19–1.24; P < 0.0001; See Additional file 1: Figure S10f).

To assess the effect that low quality papers had on the results, the 7 studies that scored < 7 using NOS were removed and then the primary and secondary outcomes were analysed a second time. The effect sizes remain the same after the removal of low-quality studies (See Additional file 1: Figure S11).

## Discussion

In this systematic review and meta-analysis of 23 studies involving over 15,000,000 participants, body weight variability was associated with significantly increased risk of cardiovascular morbidity and mortality. This risk remains present regardless of ethnicity or diabetic status.

A previous meta-analysis performed by Zou et al*.* [34] also observed a similar significantly increased risk of CVD deaths (RR = 1.36; 95% CI 1.22–1.52; P < 0.0001) and CVD events (RR = 1.49; 95% CI 1.26–1.76; P < 0.0001) associated with body-weight variability, however no analysis on the risk of other cardiovascular outcomes or the effect of ethnicity or diabetes status on CV risk was performed. Our meta-analysis further builds upon this previous study by analysing the summative risk of cardiovascular outcomes associated with a + 1 SD increase in body weight variability. Our analysis also includes 16 studies published since 2018 that were not present in this previous meta-analysis, providing an additional 14,945,638 participants in total [4, 6, 8, 10, 12, 15–22, 25, 27].

The results between sub-groups of ethnicities are remarkably consistent. However, this study has only White Europeans and East Asians and as such data on different ethnicities is required. A recent study of individuals living with T2D in Scotland found that ethnically Pakistani individuals had a significantly higher risk of CVD than their White counterparts [35]. A similar study found that South Asians with T2D had a higher risk of experiencing MI than Caucasians [36]. The American Heart Association has reported that African Americans have the highest risk of CVD relative to other ethnic populations, with ~ 47% of African American individuals over the age of 20 suffering from at least one form of CVD [37]. Future studies investigating weight variability and associated CV risk should focus these at-risk populations in order to address this gap in the data and investigate whether this observed risk is robust in these ethnicities.

Obesity is a major risk factor for type 2 diabetes, cardiovascular disease, and death [38–40]. Therefore, weight loss is recommended, and it has been shown to produce significant improvements in cardio-metabolic health [41]. However, maintaining the lost weight is challenging and this is usually followed by progressive regain [42]. The effect of weight cycling to health is controversial. Our meta-analysis showed that body weight variability was associated with a significant increase in the risk of any cardiovascular events and MI independent of diabetes status and ethnicity. It also found that weight variability is significantly associated with increased risk of stroke in individuals with diabetes. This could have implications specific to the treatment plans of individuals who are at increased risk of CV events. In addition, this adds to the growing body of evidence that treatment plans promoting weight loss may need to consider weight cycling too. However, as observational data was used in this analysis, causality cannot be established between weight variability and CVD. The use of genomic techniques such as mendelian randomisation may be useful in establishing future causality.

The biological mechanism by which body weight variability may increase the risk of CVD is uncertain. Some have suggested that this variability may increase oxidative stress and generate a low-level inflammation which [43], as the link between inflammation and certain CV diseases such as atherosclerosis is well established [44], this could increase the risk of CVD. Others have suggested an epigenetic link, as weight cycling has been shown cause the up regulation of genes associated with clotting and cardiomyopathy [45]. However, the exact mechanism for how weight variability may lead to CVD is still unclear. Investigating the biological mechanism and identifying biomarkers of weight variability could be crucial in identifying those individuals most at risk of CVD.

Weight variability itself may not cause CV disease per se, rather it could be a consequence of pre-existing illnesses that have worse prognoses. People with diabetes are previously shown to experience greater weight variability than people without diabetes [46]. In addition, weight change has been shown to vary by race/ethnicity [47]. However, sensitivity analysis by diabetes status and ethnicity yielded largely similar results.

### Limitations

This study had several limitations. The studies included in this analysis used various definitions and metrics to capture weight variability, which make comparison between studies difficult. However, subgroup analysis has found that the association between weight variability and CVD remains significant regardless of the metric used. A further limitation is the grouping of relative risk estimates from studies that compared different quantiles. However, our sensitivity analysis found statistically significant associations when we compared quantiles with homogenous definition of strata. This meta-analysis was also performed on observational data, and as such a causative link between weight variability and CVD cannot be made. Furthermore, when analysing the effect of diabetes status on the association between weight variability and CVD, we assumed that unless it was explicitly stated in the paper that the study was conducted in people with diabetes, they were non-diabetic. This study also did not consider whether weight loss was intentional or unintentional, which may add confounding variables as some unintentional weight variability in participants may be caused by an underlying pathology that may simultaneously negatively affect the participants cardiovascular health. While there are studies that adjust for baseline blood pressure and lipid levels, others did not (See Additional file 1: Table S3).

## Conclusions

This systematic review and meta-analysis has found that body weight variability is associated with an increased risk of cardiovascular events, cardiovascular death, myocardial infarction, stroke, and compound CVD outcomes independent of diabetes status and ethnicity, however a large degree a heterogeneity between studies was observed in these analyses. More research is needed to prove causation and investigate the mechanisms responsible for this effect.

## Supplementary Information

**Additional file 1: Appendix S1.** Search Strategies. **Appendix S2.** Description of Emails. **Appendix S3. **List of Inclusion and Exclusion Criteria. **Table S1.** Descriptions and Definitions of Weight Variability Metrics. **Table S2. **Table of Study Characteristics. **Table S3.** Additional Table of Study Characteristics. **Figure S1.** Results of Per +1 SD in Weight Variability Analysis. **Figure S2.** Results of Degree of BMI Variability Analysis. **Figure S3.** Results of Ethnicity Stratification. **Figure S4.** Results of Diabetes Status Stratification. **Figure S5.** Results of Metric of Variability Stratification. **Figure S6.** Results of Quantile Stratification. **Figure S7**. Results of Previous Cardiovascular Disease Stratification. **Figure S8.** Results of adjustment for change in BMI or average BMI stratification. **Figure S9.** Results of Univariate Meta-Regression by Age. **Figure S10.** Egger’s Regression and Funnel Plots. **Figure S11.** Results of Newcastle-Ottawa Bias Analysis. **Appendix S4.** MOOSE Checklist. **Table S4.** Newcastle – Ottawa Scale Quality Assessment Results. **Appendix S5.** Newcastle-Ottawa Quality Assessment Scale.

## Data Availability

All data analysed during this study can be found in the included published articles. The datasets generated during the current study are available from the corresponding author on reasonable request.

## Abbreviations

ASV: Average successive variation
BMI: Body mass index
CIs: Confidence Intervals
CoV: Coefficient of variation
CV: Cardiovascular
CVD: Cardiovascular disease
MI: Myocardial infarction
MOOSE: Meta-analyses of observational studies in epidemiology
NOS: Newcastle- Ottawa quality assessment scale for cohort studies
RMSE: Root mean squared error of the residual variation
RR: Relative risk
SD: Standard deviation
SE: Standard ERROR
T2D: Type II diabetes mellitus
VIM: Variability independent of the mean
