# Supplementary material for: Weight variability and cardiovascular outcomes: a systematic review and meta-analysis
Source: Cardiovasc Diabetol. 2023 Jan 9;22:5. doi: 10.1186/s12933-022-01735-x (PMC9830835; doi:10.1186/s12933-022-01735-x)
Supplement: Supplementary file 1 — Additional file 1: Appendix S1. Search Strategies. Appendix S2. Description of Emails. Appendix S3. List of Inclusion and Exclusion Criteria. Table S1. Descriptions and Definitions of Weight Variability Metrics. Table S2. Table of Study Characteristics. Table S3. Additional Table of Study Characteristics. Figure S1. Results of Per +1 SD in Weight Variability Analysis. Figure S2. Results of Degree of BMI Variability Analysis. Figure S3. Results of Ethnicity Stratification. Figure S4. Results of Diabetes Status Stratification. Figure S5. Results of Metric of Variability Stratification. Figure S6. Results of Quantile Stratification. Figure S7. Results of Previous Cardiovascular Disease Stratification. Figure S8. Results of adjustment for change in BMI or average BMI stratification. Figure S9. Results of Univariate Meta-Regression by Age. Figure S10. Egger’s Regression and Funnel Plots. Figure S11. Results of Newcastle-Ottawa Bias Analysis. Appendix S4. MOOSE Checklist. Table S4. Newcastle – Ottawa Scale Quality Assessment Results. Appendix S5. Newcastle-Ottawa Quality Assessment Scale. [file 12933_2022_1735_MOESM1_ESM.docx]

**Weight Variability and Cardiovascular Outcomes: A Systematic Review and Meta-Analysis**

Robert J Massey^1^, Moneeza K Siddiqui^1^, Ewan R Pearson^1^, Adem Y Dawed^1^*

^1^Population Health & Genomics, University of Dundee, UK.

**Supplementary Material**

# Table of Contents

[Table of Contents 2](#_Toc120028478)

[Appendix 1: Search Strategies 3](#_Toc120028479)

[Appendix 2: Description of Emails 4](#_Toc120028480)

[Appendix 3: List of Inclusion and Exclusion Criteria 5](#_Toc120028481)

[Table S1: Descriptions and Definitions of Weight Variability Metrics 6](#_Toc120028482)

[Table S2: Table of Study Characteristics 7](#_Toc120028483)

[Table S3: Additional Table of Study Characteristics 14](#_Toc120028484)

[Figure S1: Results of Per +1 SD in Weight Variability Analysis 22](#_Toc120028485)

[Figure S2: Results of Degree of BMI Variability Analysis 24](#_Toc120028486)

[Figure S3: Results of Ethnicity Stratification 26](#_Toc120028487)

[Figure S4: Results of Diabetes Status Stratification 32](#_Toc120028488)

[Figure S5: Results of Metric of Variability Stratification 37](#_Toc120028489)

[Figure S6: Results of Quantile Stratification 47](#_Toc120028490)

[Figure S7: Results of Previous Cardiovascular Disease Stratification 51](#_Toc120028491)

[Figure S8: Results of adjustment for change in BMI or average BMI stratification 57](#_Toc120028492)

[Figure S9: Results of Univariate Meta-Regression by Age 61](#_Toc120028493)

[Figure S10: Egger’s Regression and Funnel Plots 66](#_Toc120028494)

[Figure S11: Results of Newcastle-Ottawa Bias Analysis 72](#_Toc120028495)

[Appendix 4: MOOSE Checklist 78](#_Toc120028496)

[Table S4: Newcastle – Ottawa Scale Quality Assessment Results 80](#_Toc120028497)

[Appendix 5: Newcastle-Ottawa Quality Assessment Scale 82](#_Toc120028498)

[References 83](#_Toc120028499)

# Appendix 1: Search Strategies

**WEB OF SCIENCE**

(ALL=(Fluctuation) OR ALL=(Oscillation) OR ALL=(Variation)) AND (ALL=(Body Mass Index) OR ALL=(Body Weight)) AND (ALL=(Cardiovascular Disease) OR ALL=(Cardiovascular Outcomes))

**PUBMED**

#1 “Fluctuation”[tw] OR “Oscillation”[tw] OR “Variation”[tw]

#2 “Body Weights and Measures”[Mesh] OR “Body Mass Index”[tw] OR “Body Weight”[tw]

#3 "Cardiovascular Diseases"[Mesh] OR “Cardiovascular Disease”[tw] OR “Cardiovascular Outcomes”[tw]

#4 #1 AND #2 AND #3

**COCHRANE LIBRARY**

#1 (Fluctuation):ti,ab,kw OR (Oscillation):ti,ab,kw OR (Variation):ti,ab,kw

#2 [mh "Body Weights and Measures"] (Body Mass Index):ti,ab,kw OR (Body Weight):ti,ab,kw

#3 [mh "Cardiovascular Diseases"] (Cardiovascular Disease):ti,ab,kw OR (Cardiovascular Outcomes):ti,ab,kw

#4 #1 AND #2 AND #3

# Appendix 2: Description of Emails

“Dear [PRIMARY AUTHOR],

My name is Robert Massey. I am a postgraduate researcher at the University of Dundee, Scotland. I am investigating the impact of bodyweight-fluctuation on cardiovascular health.

I have recently read the abstract for your paper, "[TITLE OF PAPER]". It appears to be potentially very useful to my research. Unfortunately, I cannot find a full-text copy of the paper online or through my library. As such, I am writing to ask whether it would be possible for you to send me an electronic reprint, if you have a copy?

Thank you in advance for your time.

All the best,

Robert Massey

# Appendix 3: List of Inclusion and Exclusion Criteria

1. Studies must have investigated the association between weight variabilitys and subsequent CVD.
2. Participants must be at least 18 years old.
3. The studies included must have included at minimum 500 total participants.
4. The studies included must have had a follow up period of at least 1 year.
5. The studies included must have published relative risk estimates such as risk ratios, rate ratios, odds ratios, or hazard ratios with associated 95% confidence intervals (CIs) for recorded events.
6. Studies not published in English were excluded.

# Table S1: Descriptions and Definitions of Weight Variability Metrics

| **Descriptions and Definitions of Weight Variability Metrics** | | | |
| --- | --- | --- | --- |
| **Measure of Variability** | **Definition** | **Formula** | **Studies Utilising Metric** |
| Coefficient of Variation | The standard deviation divided by the mean. | $CoV= \frac{\sigma}{\mu}$  where:  $\mu=$ population mean,  $\sigma=$ population standard deviation | Aucott *et al*., 2016 (1); Lissner *et al*., 1991 (2); Nam *et al*., 2019 (3) |
| Average Successive Variability | The average absolute difference between  successive values. | $ASV =\frac{\left( x_{1}-x_{2} \right)+\ldots+(x_{n-1}-x_{n})}{n-1}$  where:  *n* = the number of measurements | Bangalore et al., 2017 (4); Bangalore et al., 2018 (5); Choi et al., 2019 (6); Li et al., 2021 (7); Yeboah et al., 2019 (8); Youk et al., 2020 (9) |
| Root Mean Squared Error of Residual Variation | The square root of the mean of the squared residuals (different to SD as model is predictive). | $RMSE= \sqrt{\frac{1}{n}\sum_{i=1}^{n} {(y_{i}-\hat{y}_{i})}^{2}}$  where:  $y_{i}=$ observed values,  $\hat{y}_{i}=$expected values,  $n=$ number of observations | Cologne et al., 2019 (10); Dyer et al., 2000 (11) |
| Standard Deviation | The square root of the mean of the squared residuals. | $SD= \sqrt{\frac{1}{n}\sum_{i=1}^{n} {(y_{i}-\bar{y})}^{2}}$  where:  $y_{i}=$ observed values,  $n=$ number of observations | Ceriello et al., 2021 (12) |
| Variability Independent of the Mean | A method by which SD is de-correlated from the mean as to be independent from it. | $VIM=\frac{100 \times\sigma}{\bar{y}^{2}}$  where:  $y=$ the observed values,  $\sigma=$ the standard deviation | Kim et al., 2020 (13); Kim et al., 2021 (14); Lee et al., 2020 (15); Lee et al., 2020 (16); Nam et al., 2020 (17) |

**Table S1: Definitions and descriptions of the metrics used to capture weight/BMI Variability from the papers included in the meta-analysis.**

#

# Table S2: Table of Study Characteristics

| **Study** | **PMID** | **Total pop.** | **Av.^*^ Age (years)** | **Av.* Weight (kgs)** | **Av.* BMI** | **Av.* Follow-up (years)** | **Pop. Sex**  **(% Male)** | **Pop. Ethnicity (Majority)** | **Metric of Variability** | **BMI or Weight Variability** | **Population Description** |
| --- | --- | --- | --- | --- | --- | --- | --- | --- | --- | --- | --- |
| **Aucott, L. S. (2016)** | 27466237 | 29,316 | 58 | 92.5 | 33.2 | 5.2 | 54.4 | White | Coefficient of Variation | Weight | Patients with BMI > 25 and incident diabetes diagnosed between 2002 and 2006 |
| **Bangalore, S. (2017)** | 28691788 | 9509 | 61.8 | 84.7 | *Not Reported* | 4.9 | 81.0 | White | Average Successive Variability | Weight | Patients with clinically evident coronary artery disease and levels of low-density lipoprotein cholesterol below 130 mg per decilitre (3.4 mmol per litre) who had been randomly assigned to receive either 10 mg or 80 mg of atorvastatin per day |
| **Bangalore, S. (2018)** | 30571333 | 6408 | 61.7 | 85.1 | 29.2 | CARDS = 3.9; ASPEN = 4.0; TNT = 4.9 | 68.3 | White | Average Successive Variability | Weight | Patients with type 2 diabetes mellitus at baseline, who were enrolled in either the CARDS, ASPEN, or TNT clinical trials of statins |
| **Ceriello, A. (2021)** | 34446018 | 100,576 | Q1 = 66.0; Q2 = 65.0; Q3 = 64.0; Q4 = 62.0 | Q1 = 81.3; Q2 = 85.0; Q3 = 88.0; Q4 = 91.1 | Q1 = 28.1; Q2 = 29.0; Q3 = 29.6; Q4 = 30.7 | 4.4 | Q1 =52.9; Q2 = 56.1; Q3 = 57.5; Q4 = 56,0 | White | Standard Deviation | Weight | Individuals without established CVD |
| **Cho, I. J. (2017)** | 29216261 | 379,535 | 51.7 | *Not Reported* | 24.0 | 10.7 | 56 | East Asian | Categorical | BMI | Adults over 40 years of age without pre-existing CV disease or cancer at baseline |
| **Choi, D. (2019)** | 31266987 | 240,640 | Q1 = 55.4; Q2 = 55.9; Q3 = 56.1; Q4 = 56.6; Q5 = 57.7 | *Not Reported* | Q1 = 23.7; Q2 = 23.7; Q3 = 23.9; Q4 = 24.1; Q5 = 24.6 | 7 | Q1 = 63.7; Q2 = 58.1; Q3 = 58.5; Q4 = 57.1; Q5 = 51.1 | East Asian | Average Successive Variability | Weight | Adults > 40 years. |
| **Cologne, J. (2019)** | 30874785 | 3779 | 35.0 | *Not Reported* | 22.1 | 27 | 30.1 | East Asian | Root Mean Squared Error of Residual Variation | BMI | Atomic bomb survivors. |
| **Diaz, V. A. (2005)** | 15847242 | 8479 | SNO = 44.7; SO = 47.6; WG = 38.9; WL = 51.6; WF = 43.8 | *Not Reported* | SNO = 24.0; SO = 32.8; WG = 24.6; WL = 30.8; WF = 26.8 | 21 | SNO = 51.2; SO = 48.5; WG = 41.0; WL = 31.0; WF = 39.6 | White | Categorical | BMI | United States Civilians enrolled in The National Health and Nutrition Examination Survey I who were 25–74 years old at the time of the index interview  (1971–1974). |
| **Dyer, A. R. (2000)** | 10968377 | 1281 | 55.3 | *Not Reported* | 25.8 (3.1) | 25 | 100 | White | Root Mean Squared Error of Residual Variation | Weight | Men who had been employed at Chicago Western Electric Company’s Hawthorne works in Chicago, Illinois, for at least 2 years and who were aged 40–55 years in 1957. |
| **Jeong, S. (2021)** | 33980955 | 67,101 | WG = 51.3;  NWC = 50.5;  WL = 51.2 | *Not Reported* | WG = 27.1;  NWC = 26.9;  WL = 28.1 | 7 | WG = 50.5;  NWC = 63.2;  WL = 50.6 | East Asian | Categorical | Weight | Obese adults from the Korean National Health Insurance Service who received health examinations in three separate biennial periods. |
| **Kim, D. (2020)** | 33397045 | 4,244,460 | Q1 = 46.1; Q2 = 45.5; Q3 = 44.5; Q4 = 42.2 | Q1 = 46.1; Q2 = 45.5; Q3 = 44.5; Q4 = 42.2 | Q1 = 23.7; Q2 = 23.6; Q3 = 23.6; Q4 = 23.7 | 4.4 | Q1 = 71.7; Q2 = 69.7; Q3 = 69.2; Q4 = 63.0 | East Asian | Variability Independent of the Mean | Weight | South Koreans enrolled in the Korean National Health Insurance Service. |
| **Kim, M. N. (2021)** | 33911167 | 726,736 | Q1 = 49.74; Q2 = 48.31; Q3 = 47.17; Q4 = 44.88 | Q1 = 77.7; Q2 = 78.2; Q3 = 79.0; Q4 = 80.8 | Q1 = 27.53; Q2 = 27.61; Q3 = 27.81; Q4 = 28.47 | 8.1 | Q1 = 86.1; Q2 = 86.9; Q3 = 86.1; Q4 = 81.3 | East Asian | Variability Independent of the Mean | Weight | South Koreans enrolled in the Korean National Health Insurance Service diagnosed with non-alcoholic fatty liver disease. |
| **Lee, H. J. (2020)** | 31585180 | 8,091,401 | 48.1 | 64.4 | *Not Reported* | 7.8 | 58.7 | East Asian | Variability Independent of the Mean | Weight | South Koreans enrolled in the Korean National Health Insurance Service without a history of atrial fibrillation. |
| **Lee, H. J. (2020)** | 32534567 | 670,797 | 57.80 | 66.52 | 24.97 | 7.0 | 64.9 | East Asian | Variability Independent of the Mean | Weight | South Koreans enrolled in the Korean National Health Insurance Service without a history of atrial fibrillation, but diagnosed with type II diabetes. |
| **Li, Y. (2021)** | 34195237 | 1691 | 72 | 90.7 | *Not Reported* | 3.5 | 50.5 | White | Average Successive Variability | Weight | Patients with heart failure with preserved ejection fraction from the Americas from the Treatment of Preserved Cardiac Function Heart Failure with an Aldosterone Antagonist trial. |
| **Lissner, L. (1991)** | 2041550 | 3171 | Men = 42.8; Women = 43.5 | Men = 77.0; Women = 63.2 | Men = 25.9; Women = 24.9 | 32 | 43.1 | White | Coefficient of Variation | Weight | Initially CHD free residents of Framingham, Massachusetts. |
| **Merz, C. N. B. (2018)** | 30507935 | 795 | *Not Reported* | *Not Reported* | WC = 31.8; NWC = 28.8 | 6 | 0 | White | Categorical | Weight | Women with suspected ischemia undergoing clinically indicated coronary angiography. |
| **Nam, G. E. (2019)** | 32641375 | 125,391 | Q1 = 46.7; Q2 = 45.5; Q3 = 45.1; Q4 = 45.1 | Q1 = 64.2; Q2 = 65.0; Q3 = 64.6; Q4 = 63.5 | Q1 = 23.6; Q2 = 23.7; Q3 = 23.6; Q4 = 23.6 | 7 | Q1 = 59.9; Q2 = 66.3; Q3 = 64.0; Q4 = 56.0 | East Asian | Coefficient of Variation | BMI & Weight | Representative sample cohort enrolled in the national health examination program, conducted by the Korean National Health Insurance Service. |
| **Nam, G. E. (2020)** | 29777238 | 624,237 | 56.8 | Q1 = 68.1; Q2 = 67.1; Q3 = 66.7; Q4 = 65.4 | Q1 = 25.3; Q2 = 25.0; Q3 = 24.9; Q4 = 24.7 | MI = 7.6; Stroke = 7.7; ACM = 7.8 | Q1 = 69.6; Q2 = 67.7; Q3 = 65.7; Q4 = 60.5 | East Asian | Variability Independent of the Mean | Weight | Individuals with type 2 diabetes who underwent health examinations provided by the Korean National Health Insurance System between 2009 and 2010. |
| **Sponholtz, T. R. (2019)** | 31025893 | 2725 | VNO = 44.9; VO = 48.3 | *Not Reported* | VNO = 25.2; VO = 34.8 | 27 | VNO = 28.6; VO = 23.1 | White | Categorical | BMI | Framingham Heart Study offspring cohort. |
| **Wannamethee, S. G. (2002)** | 12456229 | 5608 | “*40 to 59*” | *Not Reported* | S = 25.5; SG = 24.7; SL = 26.6; LG = 26.8; GL = 25.4 | 8 | 100 | White | Categorical | BMI | Middle-aged men from the UK who participated in the British Regional Heart Study. |
| **Yeboah, P. (2019)** | 30553512 | 10,251 | 62.8 | *Not Reported* | 32.2 | 3.7 | 61.5 | White | Average Successive Variability | Weight | Individuals with type 2 diabetes who participated in the Action to Control Cardiovascular Risk in Diabetes (ACCORD) trial. |
| **Youk, T. M. (2020)** | 33355207 | 28,650 | *Not Reported* | *Not Reported* | DC = 24.5;  DNC = 24.2;  NDC = 24.2; NDNC = 23.7 | 13 | DC = 86.0;  DNC = 83.4;  NDC = 79.9; NDNC = 71.0 | East Asian | Average Successive Variability | BMI | Randomly selected cohort from 10% of all Koreans aged 40–80 years who had medical examination in 2002 – 2003, excluding subjects with pre-existing diseases such as cancer, hyperthyroidism, hypothyroidism, liver cirrhosis, and renal failure. |

**Table S2: A table of summary characteristics of the studies included in the final analysis.** The statistics shown in this table are reported here as they were reported in the studies they were taken from. *Av. = Average; Pop. = Population; Q_i_ = The ‘i^th^’ quantile of the population (based on weight variability); BMI = body mass index; CVD = cardiovascular disease; CHD = coronary heart disease; SNO = stable-weight, non-obese; SO = stable-weight, obese; WG = weight-gain; WL = weight-loss; WF = weight-fluctuation; WC = weight-change; NWC = no weight-change; VNO = variable weight, non-obese; VO = variable weight, obese; ACM = all-cause mortality; CARDS = Collaborative Atorvastatin Diabetes Study; ASPEN = Atorvastatin Study for Prevention of Coronary Heart Disease Endpoints in non-insulin-dependent diabetes mellitus; TNT = Treating to New Targets Study; S = Stable; SG = Stable-Gain; SL = Stable-Loss; LG = Loss-Gain; GL = Gain-Loss; DC = diabetic with CVD; DNC = diabetic without CVD; NDC = non-diabetic with CVD; NDNC = non-diabetic without CVD*. ** = Average is either median or mean, whichever was reported by the study.*

# Table S3: Additional Table of Study Characteristics

| **Study First Author (Year)** | **Number of Quantiles** | **Metric of Variability** | **Summary Statistics of Body Weight Variability** | **Reports Contributed by Study** | **Average number of measurements to calculate variability** | **Covariates used in maximally adjusted model** |
| --- | --- | --- | --- | --- | --- | --- |
| Aucott, L. S. (2016) | NA | Coefficient of Variation | NA | Any CV Event; Myocardial Infarction | 6 | Age, BMI, sex, smoking status, deprivation, weight change patterns, and antidiabetic medication regimes |
| Bangalore, S. (2017) | 5 | Average Successive Variability | Median ASV = 1.73kg | Any CV Event; Myocardial Infarction; Stroke; Composite Cardiovascular Outcome | 12 | Treatment, mean body weight, weight change taking directionality into account, age, sex, race, diabetes, hypertension, smoking, baseline LDL-C, baseline total cholesterol, baseline triglyceride, baseline HDL-C, CKD, CHF, and time between initial and final weight measurement. |
| Bangalore, S. (2018) | 5 | Average Successive Variability | Median ASV = 1.72kg | Any CV Event; Cardiovascular Death; Myocardial Infarction; Stroke; Composite Cardiovascular Outcome | 12 | Treatment, study, mean body weight, change in weight taking directionality into account, age, sex, race, hypertension, smoking status, chronic kidney disease, baseline levels of LDL (low-density lipoprotein) cholesterol, total cholesterol, HDL (high-density lipoprotein) cholesterol, and time between initial and final weight measurement. |
| Ceriello, A. (2021) | 4 | Standard Deviation | Q1 Mean SD = 0.9 (0.6–1.1); Q2 Mean SD = 1.7 (1.5–1.9); Q3 Mean SD = 2.6 (2.3–2.9); Q4 Mean SD = 4.5 (3.8–6.1) | Any CV Event; Myocardial Infarction; Stroke | NA (“at least 5”) | Age, gender, duration of diabetes, body weight, smoking, values of HbA1c, systolic and diastolic blood pressure, total cholesterol, HDL, LDL, triglycerides, albuminuria, eGFR, retinopathy, treatment for diabetes, hypertension, dyslipidaemia, and aspirin use. |
| Cho, I. J. (2017) | NA | Categorical | NA | Any CV Event; Cardiovascular Death | NA (“more than three”) | Age, sex, baseline systolic blood pressure, diastolic blood pressure, serum glucose, total cholesterol, smoking status, alcohol consumption, hypertension, diabetes mellitus, and baseline BMI. |
| Choi, D. (2019) | 5 | Average Successive Variability | Q1 Mean ASV = 0.24 (0.10); Q2 Mean ASV = 0.50 (0.06); Q3 Mean ASV = 0.73 (0.07); Q4 Mean ASV = 1.03 (0.11); Q5 Mean ASV = 1.91 (1.13). | Composite Cardiovascular Outcome | 3 | Age, sex, baseline body mass index, change in body mass index, household income, smoking, alcohol consumption, physical activity, systolic blood pressure, fasting serum glucose, total cholesterol, underlying cancer, and underlying cardiovascular disease. |
| Cologne, J. (2019) | 5 | Root Mean Squared Error of Residual Variation | Q1 RMSE Range = 0.113-0.451; Q2 RMSE Range = 0.452-0.587; Q3 RMSE Range = 0.588-0.721; Q4 RMSE Range = 0.722-0.939; Q5 RMSE Range = 0.940-4.085. | Any CV Event; Cardiovascular Death | NA (“at least 7”) | Year of birth, mean height during the baseline period, an indicator of having ever smoked, radiation dose to the colon, overall increase in weight, and overall decrease in weight. |
| Diaz, V. A. (2005) | NA | Categorical | NA | Any CV Event; Cardiovascular Death | 5 | age, gender, race, initial BMI, smoking status, and Charlson Comorbidity Index |
| Dyer, A. R. (2000) | 5 | Root Mean Squared Error of Residual Variation | NA | Any CV Event; Cardiovascular Death | NA (“minimum of five measurements”) | Age and smoking |
| Jeong, S. (2021) | NA | Categorical | NA | Any CV Event; Cardiovascular Death; Stroke; Composite Cardiovascular Outcome | 3 | Age, sex, household income, initial body mass index, systolic blood pressure, fasting serum glucose, total cholesterol, aspartate aminotransferase, Charlson comorbidity index, smoking, alcohol consumption, and exercise frequency |
| Kim, D. (2020) | 4 | Variability Independent of the Mean | Q1 Mean VIM = 0.7 (0.6–0.9); Q2 Mean VIM = 1.3 (1.2–1.5); Q3 Mean VIM = 1.9 (1.8–2.1); Q4 Mean VIM = 3.1 (2.7–3.9) | Any CV Event; Myocardial Infarction; Stroke | 3.6 (0.5) | Age, sex, smoking status, alcohol intake, physical activity, income, hypertension, diabetes mellitus, dyslipidaemia, chronic kidney disease, number of measurement, and baseline body mass index |
| Kim, M. N. (2021) | 4 | Variability Independent of the Mean | Q1 Mean VIM = 0.7 (0.26); Q2 Mean VIM = 1.34 (0.17); Q3 Mean VIM = 2 (0.23); Q4 Mean VIM = 3.79 (1.76) | Any CV Event; Myocardial Infarction; Stroke | NA (at least 3) | Age, sex, smoking status, alcohol consumption, physical activity, hypertension, diabetes, dyslipidaemia, chronic kidney disease, and baseline BMI |
| Lee, H. J. (2020) | 4 | Variability Independent of the Mean | NA | Any CV Event | 3.52 | baseline bodyweight, baseline height, age, sex, smoking, drinking, exercise, low income, diabetes mellitus, hypertension, dyslipidaemia, and chronic kidney disease |
| Lee, H. J. (2020) | 5 | Variability Independent of the Mean | Mean VIM = 1.98% (1.45) | Any CV Event | NA (at least 3) | baseline body mass index, age, sex, smoking, drinking, exercise, low income, hypertension, dyslipidaemia, number of oral anti-diabetic medication, insulin use, duration of diabetes, and fasting glucose. |
| Li, Y. (2021) | 2 | Average Successive Variability | Median ASV = 2.1 kg (IQR 1.4–3.1) | Any CV Event; Cardiovascular Death; Myocardial Infarction; Composite Cardiovascular Outcome | 7  (range, 2–11) | Diuretics, mean body weight, change in weight taking directionality into account, age, sex, race, smoking status, diabetes status, atrial fibrillation, peripheral arterial disease, previous hospitalization for chronic heart failure, prior myocardial infarction, known stroke, chronic obstructive pulmonary disease, New York Heart Association class, systolic blood pressure, heart rate, ejection fraction, estimated glomerular filtration rate, number of weight measurement |
| Lissner, L. (1991) | 3 | Coefficient of Variation | Mean CoV = 0.057 (0.025) | Any CV Event; Cardiovascular Death; Composite Cardiovascular Outcome | NA | Age |
| Merz, C. N. B. (2018) | NA | Categorical | NA | Composite Cardiovascular Outcome | NA | Demographic and cardiovascular risk factors. |
| Nam, G. E. (2019) | 4 | Coefficient of Variation | Q1 Mean CoV = 0.7 (0.26); Q2 Mean CoV = 1.34 (0.17); Q3 Mean CoV = 2 (0.23); Q4 Mean CoV = 3.79 (1.76) | Any CV Event; Cardiovascular Death | 3.2 | Age, sex, smoking status, alcohol consumption, physical activity, household income level, hypertension, diabetes mellitus, dyslipidaemia, and body mass index at baseline |
| Nam, G. E. (2020) | 4 | Variability Independent of the Mean | Q1 Mean VIM = 0.9 (0.5); Q2 Mean VIM = 2.0 (0.3); Q3 Mean VIM = 3.2 (0.4); Q4 Mean VIM = 6.1 (2.9) | Any CV Event; Myocardial Infarction; Stroke | NA (at least 3) | Age, sex, smoking status, alcohol consumption, physical activity, income, hypertension, dyslipidaemia, chronic kidney disease, insulin use, number of oral antidiabetic agents used, and baseline BMI |
| Sponholtz, T. R. (2019) | NA | Categorical | NA | Any CV Event; Composite Cardiovascular Outcome | NA | Age, sex, examination cycle, education, smoking status, and physical activity index. |
| Wannamethee, S. G. (2002) | NA | Categorical | NA | Any CV Event; Cardiovascular Death | 3 | Age, social class, smoking status, physical activity, initial BMI, pre-existing CVD, diabetes mellitus, cancer, and poor health |
| Yeboah, P. (2019) | 4 | Average Successive Variability | Mean ASV = 3.4 (2.4) | Any CV Event; Composite Cardiovascular Outcome | NA (at least 2) | Age, gender, race (binary), arm of the trial (treatment assignment), baseline BMI, statin use, GFR, mean SBP, mean DBP, mean LDL, mean HDL, mean HBA1C, years of diabetes, cigarette smoking status, antihypertensive medication use, baseline cardiovascular disease status, and time between initial and final weight measurement |
| Youk, T. M. (2020) | NA | Average Successive Variability | NA | Any CV Event; Cardiovascular Death; Composite Cardiovascular Outcome | 3 - 11 | NA |

**Table S3: An additional table of summary characteristics of the studies included in the final analysis.** The statistics shown in this table are reported here as they were reported in the studies they were taken from.

#

# Figure S1: Results of Per +1 SD in Weight Variability Analysis


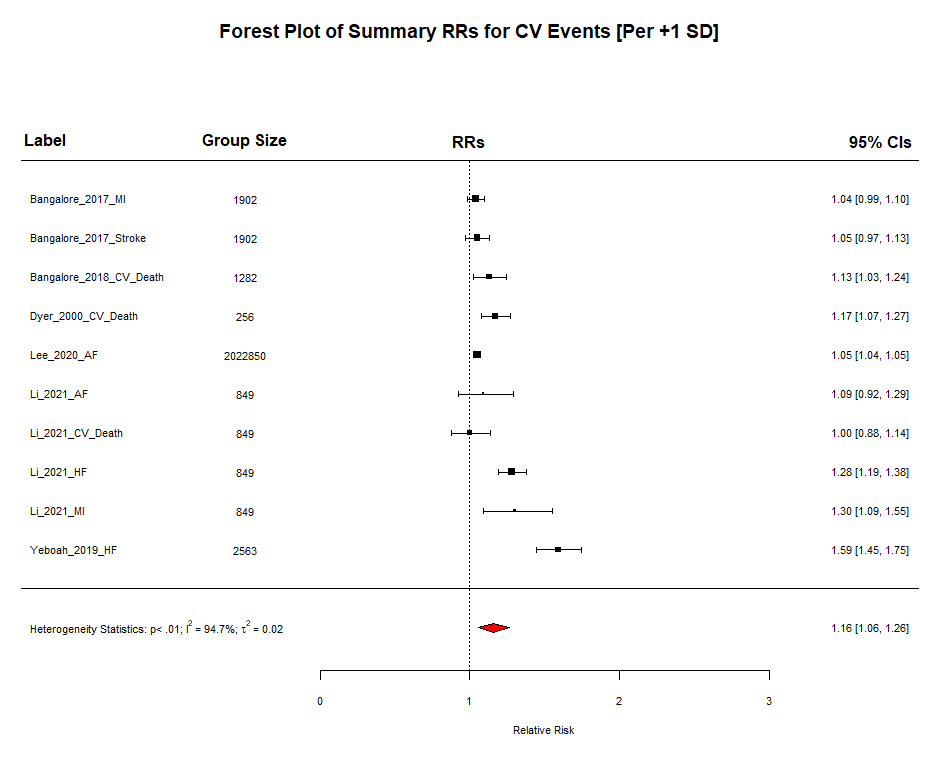


**Figure S1a: Forest plot showing the summative risk of any cardiovascular event per +1 SD increase in body weight variability.** RR = 1.16; 95% CI 1.06 – 1.26; P < 0.0001; Significant Heterogeneity (I^2^ = 94.70%; P = 0.0013).


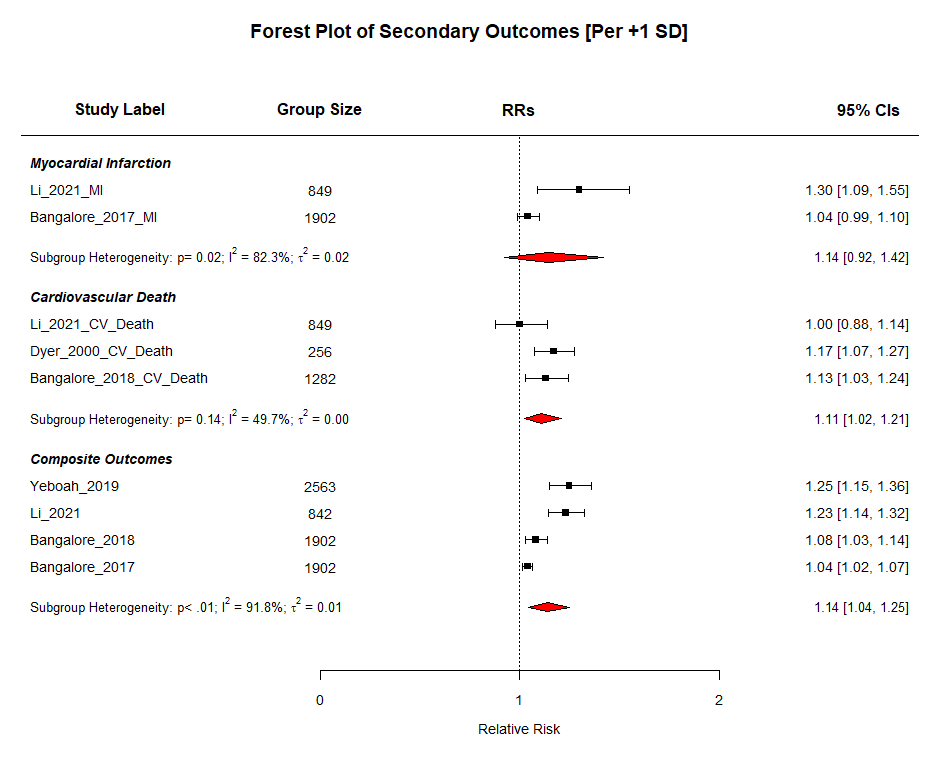


**Figure S1b: A compound forest plot showing the summative risk of the secondary outcomes per +1 SD increase in body weight variability.** The subheadings “Myocardial Infarction”, “Cardiovascular Death”, and “Composite Outcomes” are followed by the reports included in the respective sub-analysis. The number of participants in the most variable group are shown in the column “Group Size”. CV Death RR = 1.11; 95% CI 1.02 – 1.21; P = 0.0132; I^2^ = 49.66%; P for heterogeneity = 0.1359. MI RR = 1.14; 95% CI 0.92 – 1.42; P = 0.2234 ; I^2^ = 82.32%; P for heterogeneity = 0.0174. Most composite CV outcome RR = 1.14; 95% CI 1.04 – 1.25; P = 0.0047; I^2^ = 91.77%; P for heterogeneity < 0.0001.

# Figure S2: Results of Degree of BMI Variability Analysis


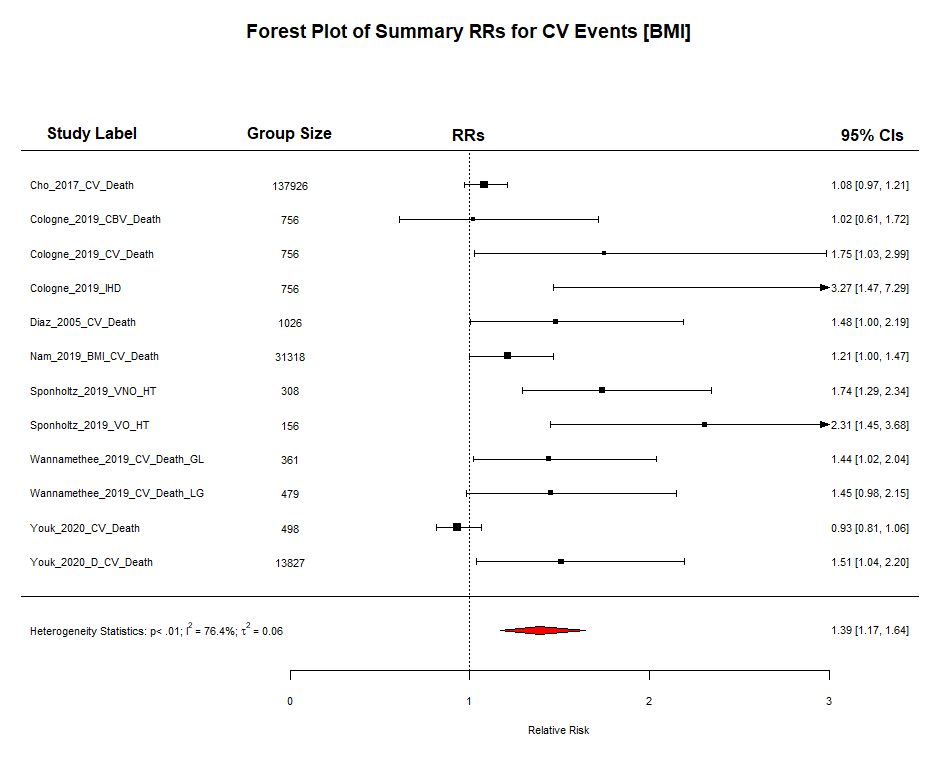


**Figure S2a: Forest plot showing the summative risk of any cardiovascular event associated with being in the top quantile of BMI variability.** RR = 1.39; 95% CI 1.17 – 1.64; P < 0.0001; Significant Heterogeneity (I^2^ = 76.39%; P < 0.0001).


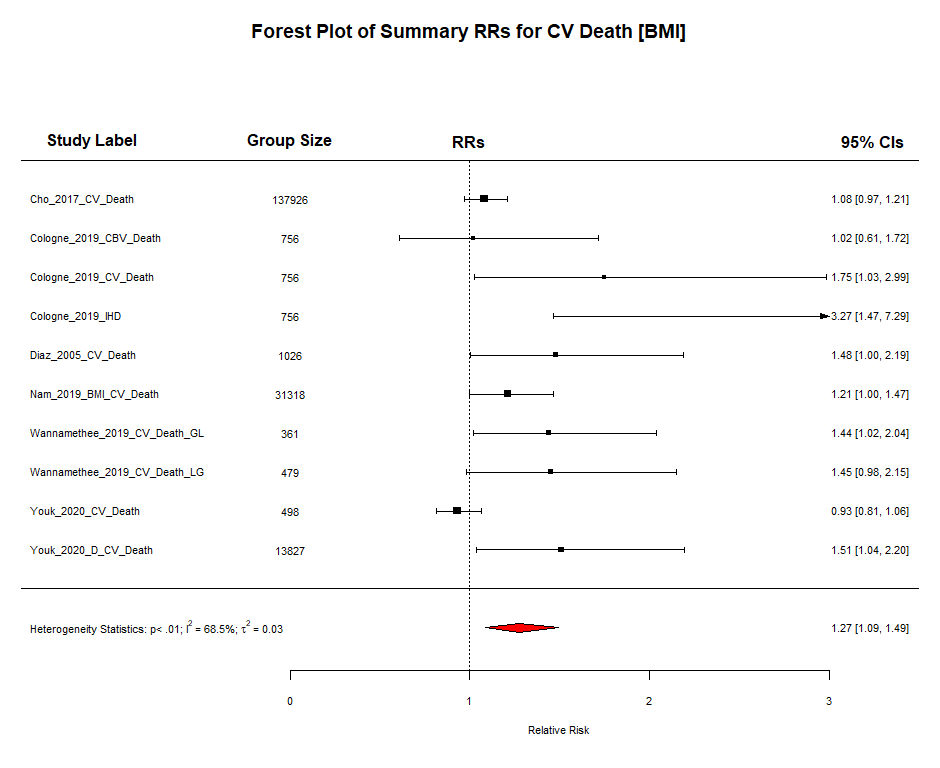


**Figure S2b: Forest plot showing the summative risk of cardiovascular death associated with being in the top quantile of BMI variability.** RR = 1.27; 95% CI 1.09 – 1.49; P = 0.0027; Significant Heterogeneity (I^2^ = 68.51%; P = 0.002).

# Figure S3: Results of Ethnicity Stratification


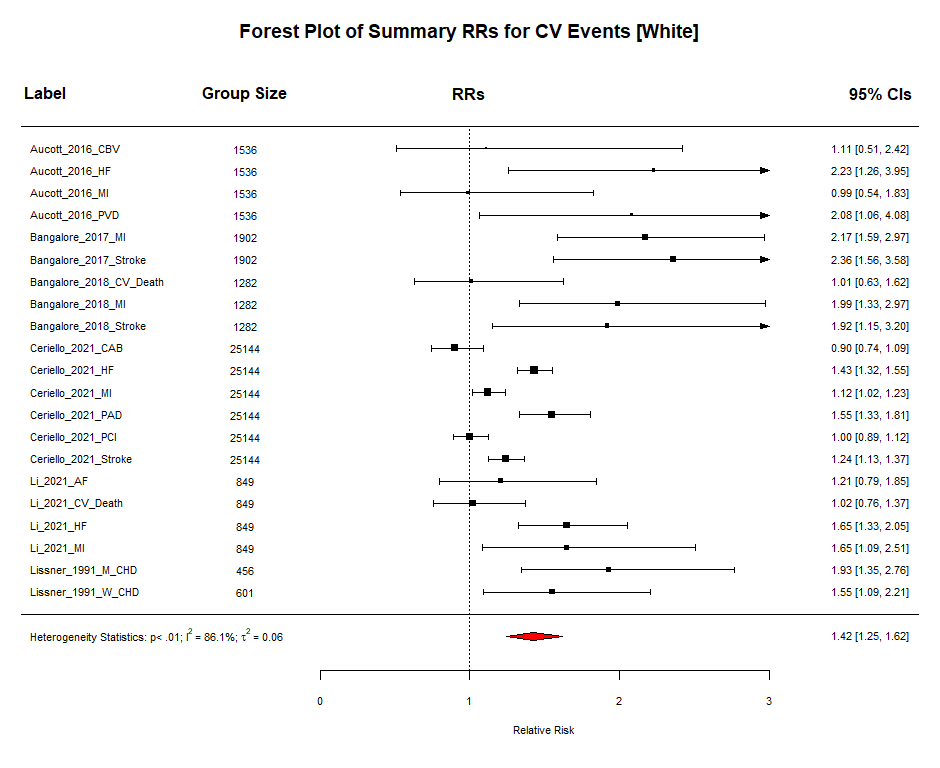


**Figure S3a: Forest plot showing the summative risk of any cardiovascular event associated with being in the top quantile of body weight variability in ethnically White individuals.** RR = 1.42; 95% CI 1.25 – 1.62; P < 0.0001; Significant Heterogeneity (I^2^ = 86.15%; P < 0.0001).


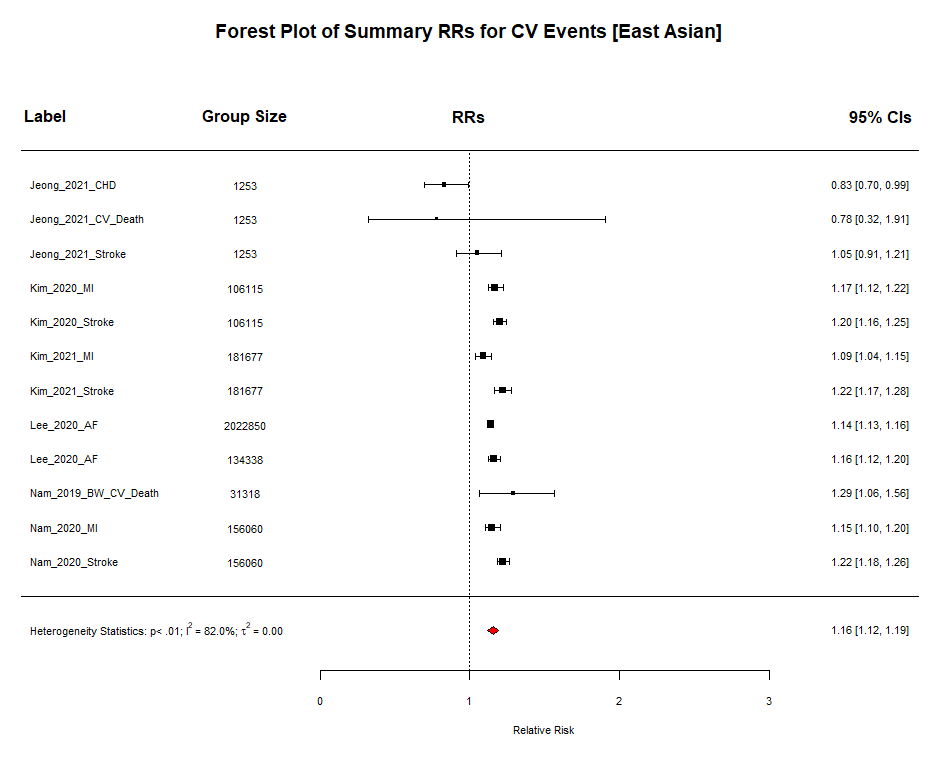


**Figure S3b: Forest plot showing the summative risk of any cardiovascular event associated with being in the top quantile of body weight variability in ethnically East Asian individuals.** RR = 1.16; 95% CI 1.12 – 1.19; P < 0.0001; Significant Heterogeneity (I^2^ = 82.02%; P < 0.0001).


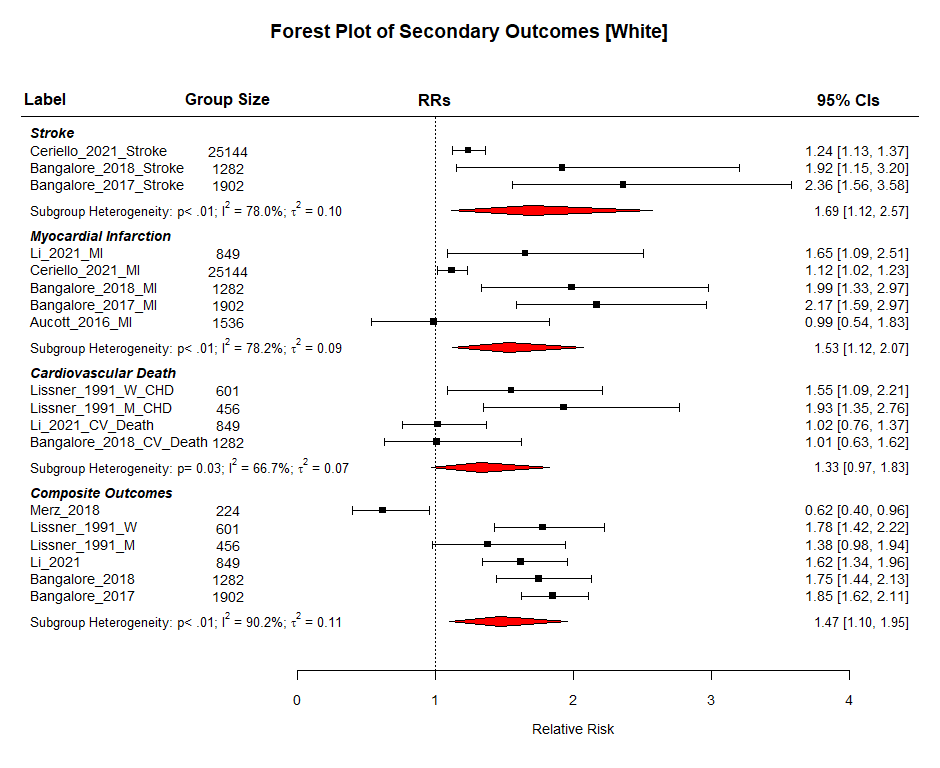


**Figure S3c:** **A compound forest plot showing the summative risk of the secondary outcomes associated with being in the most variable body weight group compared to the least variable in ethnically White individuals.** The subheadings “Stroke”, ”Myocardial Infarction”, “Cardiovascular Death”, and “Composite Outcomes” are followed by the reports included in the respective sub-analysis. The number of participants in the most variable group are shown in the column “Group Size”. CV Death RR = 1.33; 95% CI 0.97 – 1.83; P = 0.0741; I^2^ = 66.71%; P for heterogeneity = 0.0266. MI RR = 1.53; 95% CI 1.12 – 2.07; P = 0.0068; I^2^ = 78.17%; P for heterogeneity < 0.0001. Stroke RR = 1.69; 95% CI 1.12 – 2.57; P = 0.013; I^2^ = 78.02%; P for heterogeneity = 0.0039. Most composite CV outcome RR = 1.47; 95% CI 1.10 – 1.95; P = 0.0084; I^2^ = 90.21%; P for heterogeneity = 0.0002.


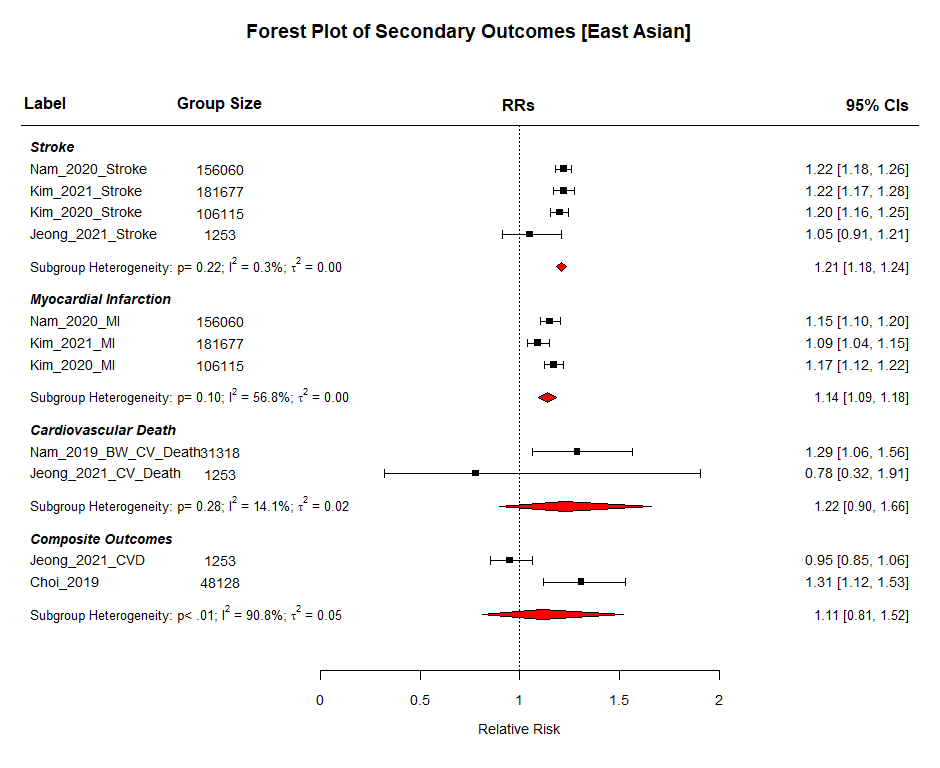


**Figure S3d: A compound forest plot showing the summative risk of the secondary outcomes associated with being in the most variable body weight group compared to the least variable in ethnically East Asian individuals.** The subheadings “Stroke”, ”Myocardial Infarction”, “Cardiovascular Death”, and “Composite Outcomes” are followed by the reports included in the respective sub-analysis. The number of participants in the most variable group are shown in the column “Group Size”. CV Death RR = 1.22; 95% CI 0.90 – 1.66; P = 0.2022; I^2^ = 14.10%; P for heterogeneity = 0.2806. MI RR = 1.14; 95% CI 1.09 – 1.18; P < 0.0001; I^2^ = 56.84%; P for heterogeneity = 0.1018. Stroke RR = 1.21; 95% CI 1.18 – 1.24; P < 0.0001; I^2^ = 0.26%; P for heterogeneity = 0.2246. Most composite CV outcome RR = 1.11; 95% CI 0.81 – 1.52; P = 0.5154; I^2^ = 90.79%; P for heterogeneity = 0.001.

# Figure S4: Results of Diabetes Status Stratification


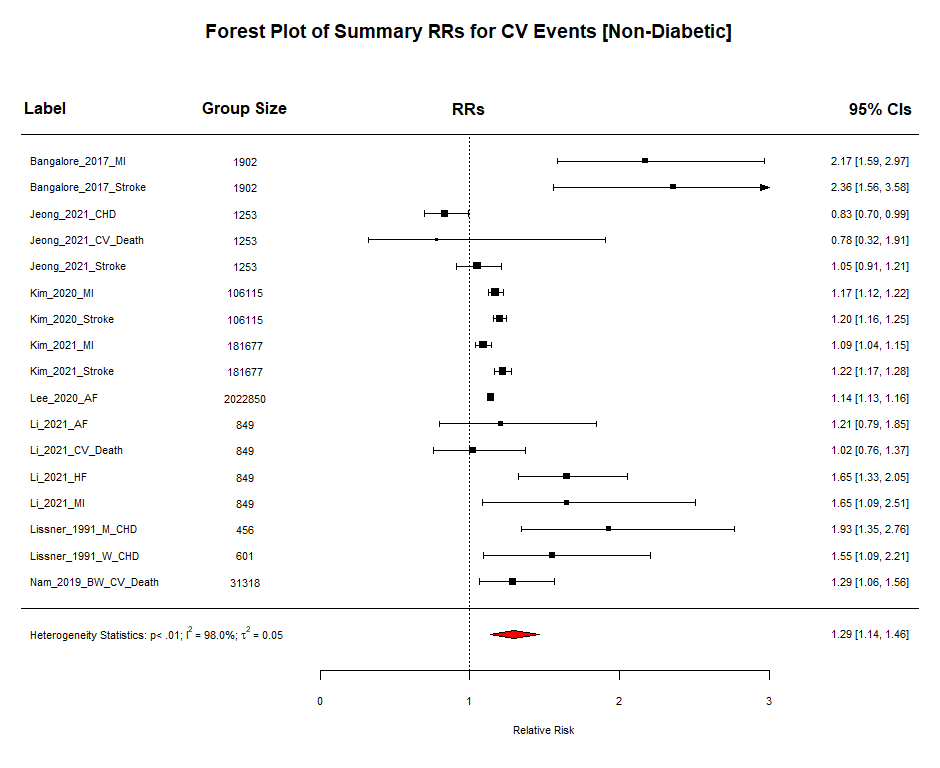


**Figure S4a: Forest plot showing the summative risk of any cardiovascular event associated with being in the top quantile of body weight variability in non-diabetic individuals.** RR = 1.29; 95% CI 1.14 – 1.46; P < 0.0001; Significant Heterogeneity (I^2^ = 98.03%; P < 0.0001).


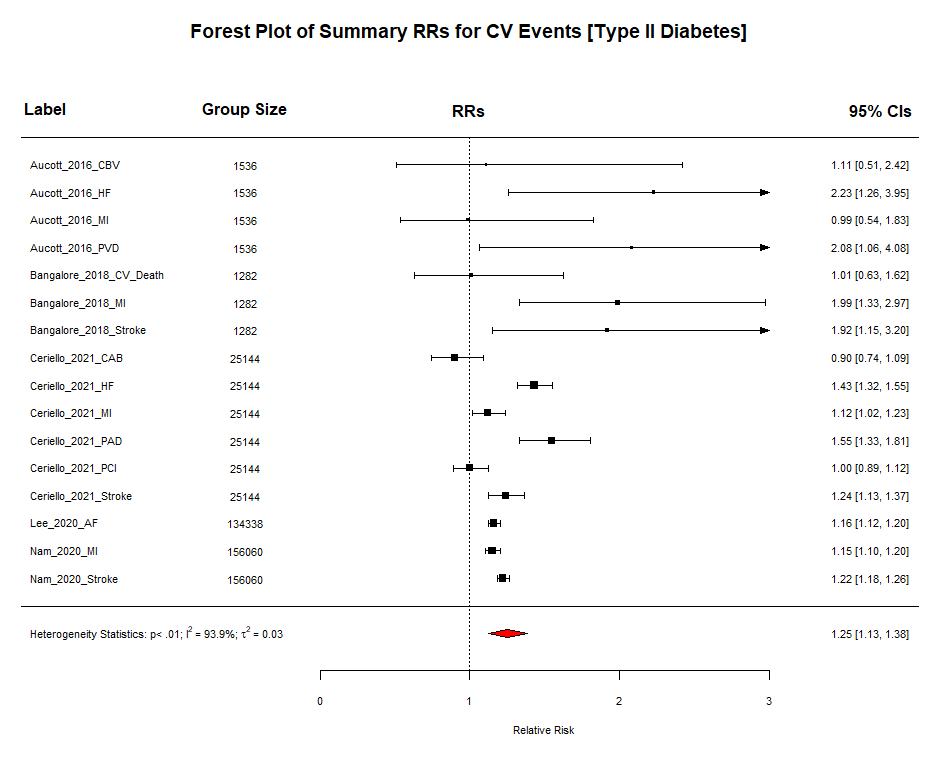


**Figure S4b: Forest plot showing the summative risk of any cardiovascular event associated with being in the top quantile of body weight variability in individuals with type II diabetes.** RR = 1.25; 95% CI 1.13 – 1.38; P < 0.0001; Significant Heterogeneity (I^2^ = 98.03%; P < 0.0001).


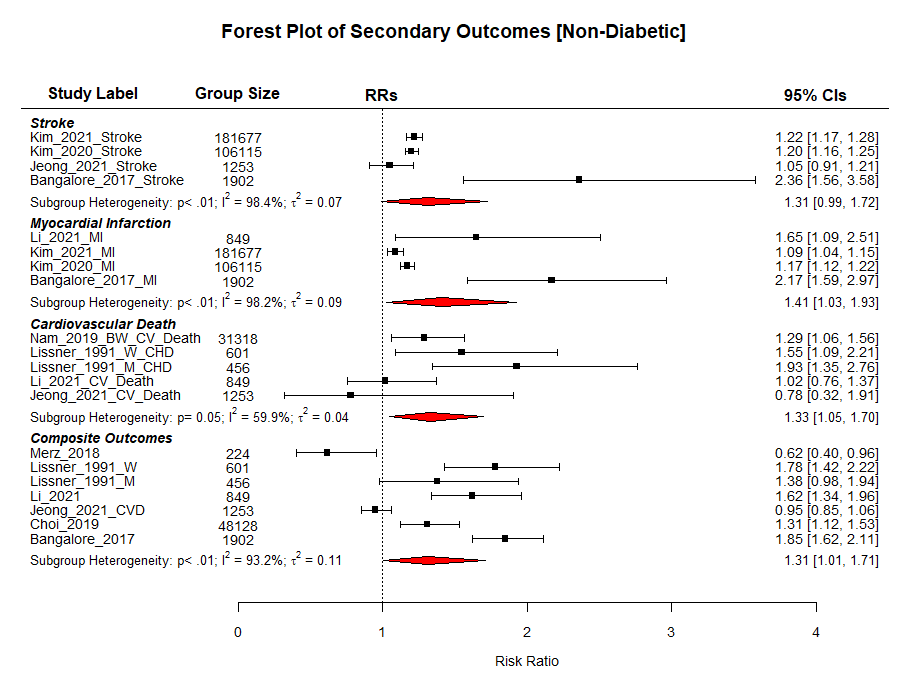


**Figure S4c: A compound forest plot showing the summative risk of the secondary outcomes associated with being in the most variable body weight group compared to the least variable in non-diabetic individuals.** The subheadings “Stroke”, ”Myocardial Infarction”, “Cardiovascular Death”, and “Composite Outcomes” are followed by the reports included in the respective sub-analysis. The number of participants in the most variable group are shown in the column “Group Size”. CV Death RR = 1.33; 95% CI 1.05 – 1.70; P = 0.0195; I^2^ = 59.94%; P for heterogeneity = 0.0523. MI RR = 1.41; 95% CI 1.03 – 1.93; P = 0.0321; I^2^ = 98.16%; P for heterogeneity < 0.0001. Stroke RR = 1.31; 95% CI 0.99 – 1.71; P = 0.056; I^2^ = 98.35%; P for heterogeneity = 0.0029. Most composite CV outcome RR = 1.31; 95% CI 1.01 – 1.71; P = 0.0442; I^2^ = 93.15%; P for heterogeneity < 0.0001.


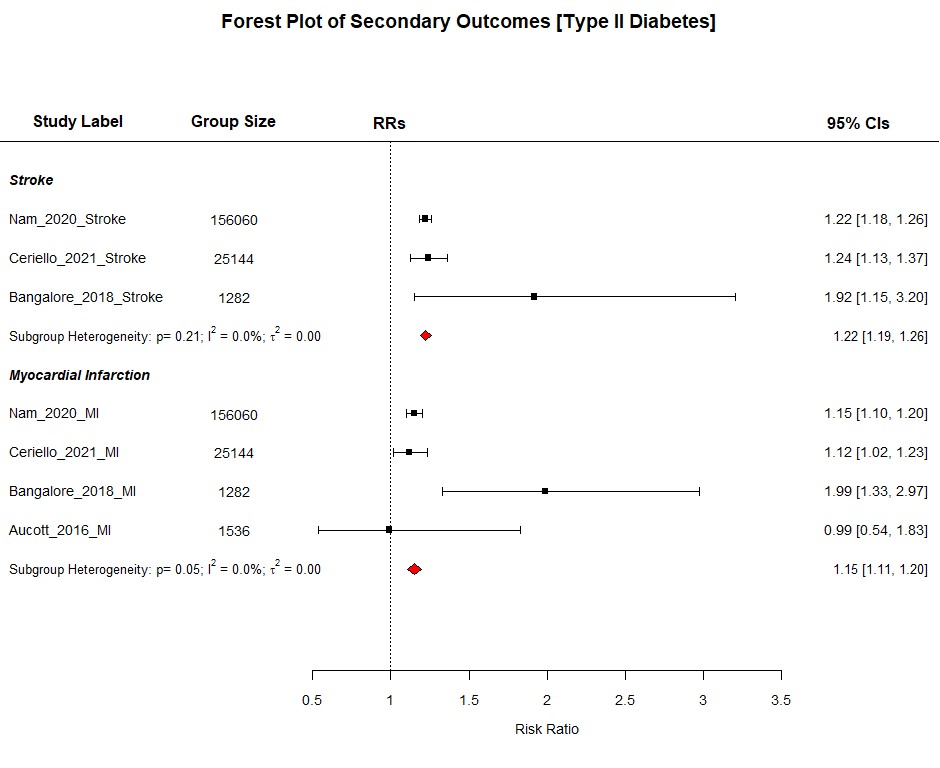


**Figure S4d: A compound forest plot showing the summative risk of the secondary outcomes associated with being in the most variable body weight group compared to the least variable in diabetic individuals.** The subheadings “Stroke” and ”Myocardial Infarction” are followed by the reports included in the respective sub-analysis. The number of participants in the most variable group are shown in the column “Group Size”. MI RR = 1.15; 95% CI 1.11 – 1.20; P < 0.0001; I^2^ = 0.00%; P for heterogeneity = 0.0533. Stroke RR = 1.22; 95% CI 1.19 – 1.26; P < 0.0001; I^2^ = 0.01%; P for heterogeneity = 0.2142.

# Figure S5: Results of Metric of Variability Stratification


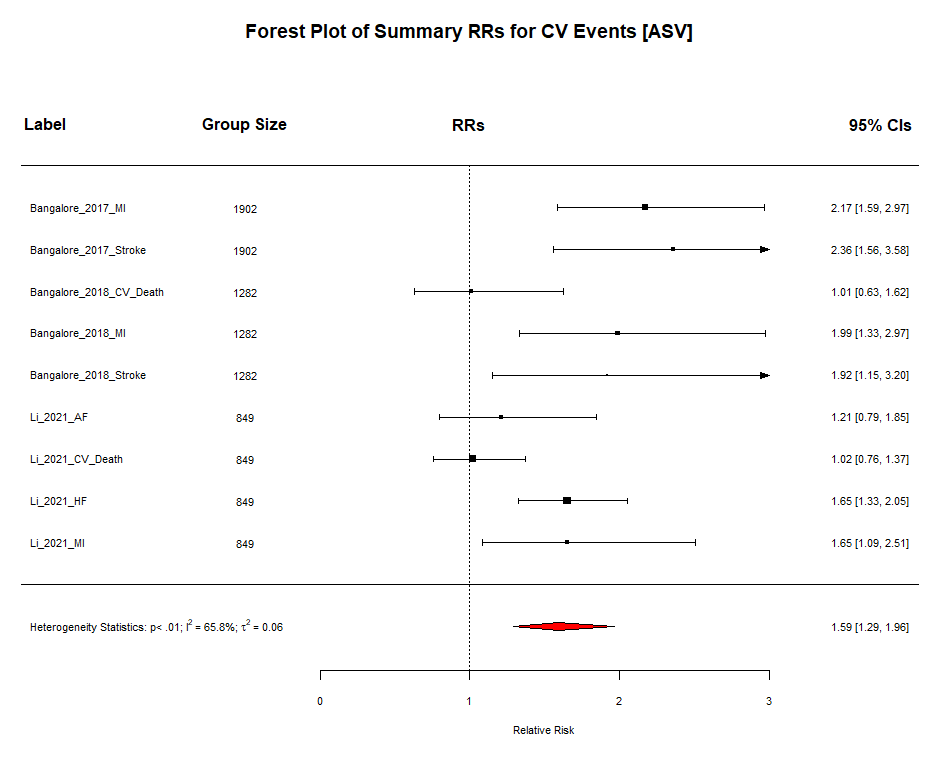


**Figure S5a: Forest plot showing the summative risk of any cardiovascular event associated with being in the top quantile of body weight variability in studies that measured variability via ASV.** RR = 1.59; 95% CI 1.29 – 1.96; P < 0.0001; Significant Heterogeneity (I^2^ = 65.75%; P = 0.003).


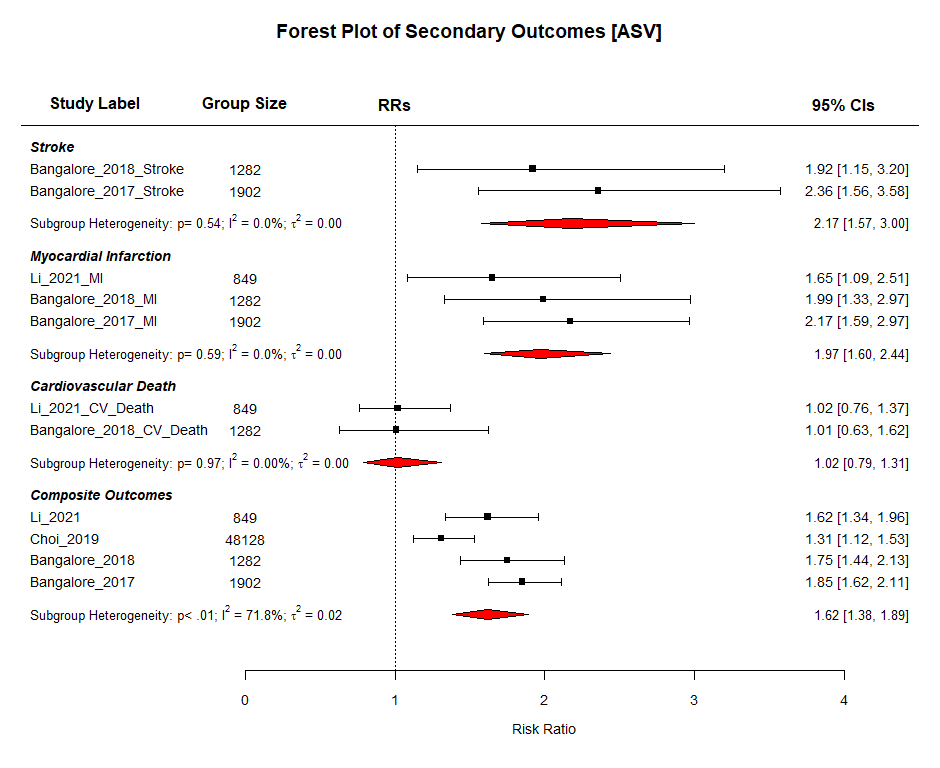


**Figure S5b: A compound forest plot showing the summative risk of the secondary outcomes associated with being in the top quantile of body weight variability in studies that measured variability via ASV.** The subheadings “Stroke”, ”Myocardial Infarction”, “Cardiovascular Death”, and “Composite Outcomes” are followed by the reports included in the respective sub-analysis. The number of participants in the most variable group are shown in the column “Group Size”. CV Death RR = 1.02; 95% CI 0.79 – 1.31; P = 0.8943; I^2^ = 0.00%; P for heterogeneity = 0.9725. MI RR = 1.97; 95% CI 1.60 – 2.44; P < 0.0001; I^2^ = 0.00%; P for heterogeneity = 0.5892. Stroke RR = 2.17; 95% CI 1.57 – 3.00; P < 0.0001; I^2^ = 0.00%; P for heterogeneity = 0.5394. Most composite CV outcome RR = 1.62; 95% CI 1.38 – 1.89; P < 0.0001; I^2^ = 71.83%; P for heterogeneity = 0.009.


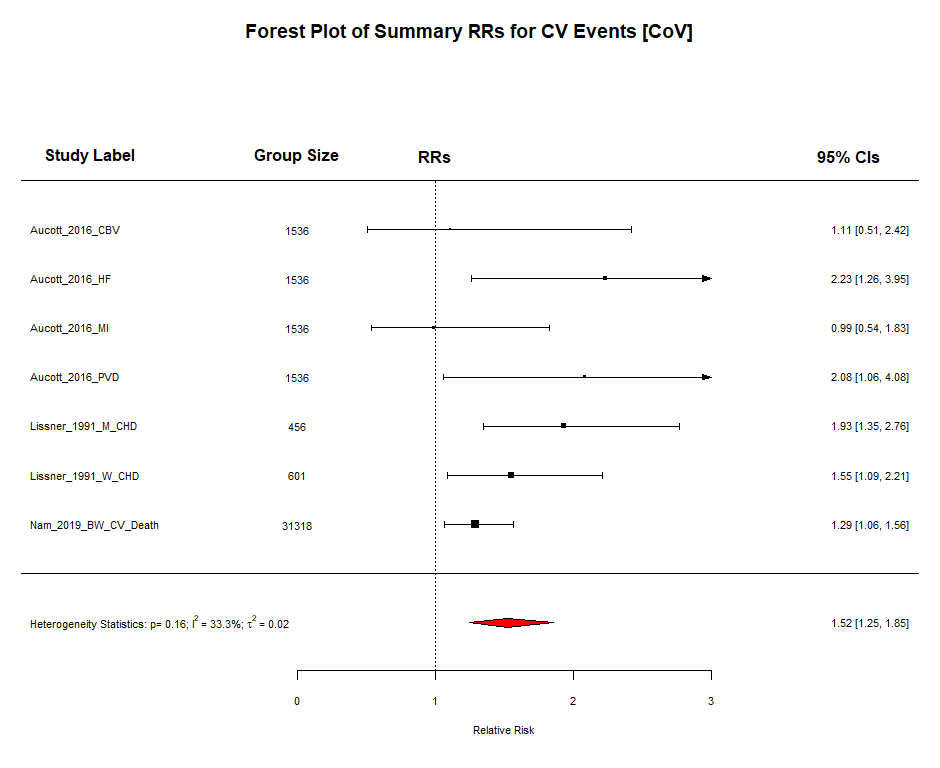


**Figure S5c: Forest plot showing the summative risk of any cardiovascular event associated with being in the top quantile of body weight variability in studies that measured variability via CoV.** RR = 1.52; 95% CI 1.25 – 1.85; P < 0.0001; Insignificant Heterogeneity (I^2^ = 33.29%; P = 0.1635).


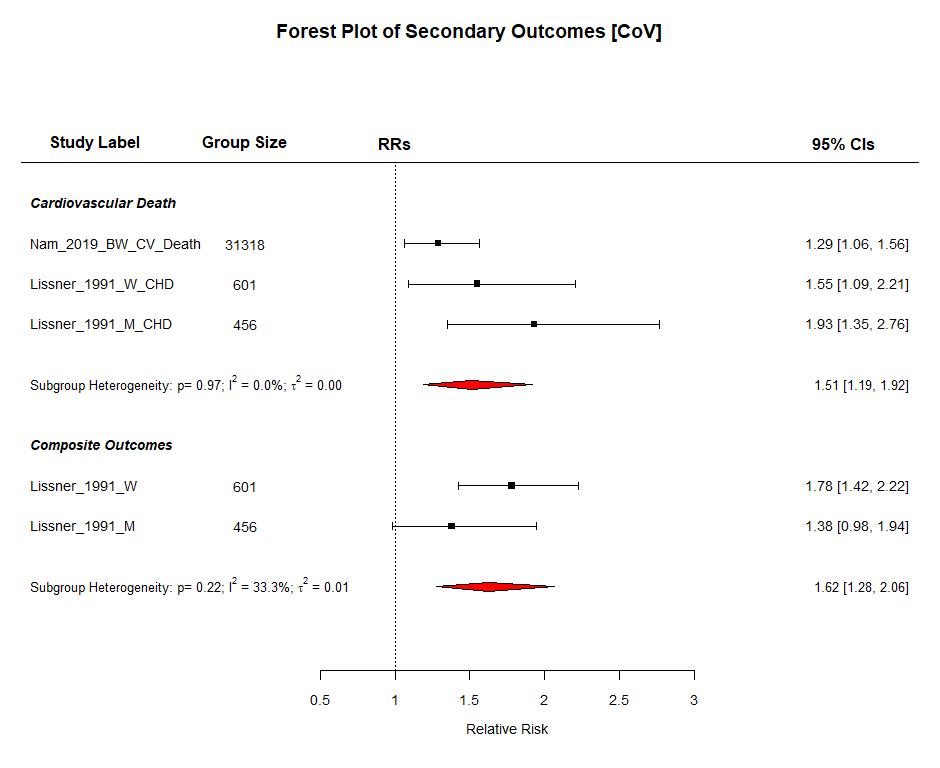


**Figure S5d: A compound forest plot showing the summative risk of the secondary outcomes associated with being in the top quantile of body weight variability in studies that measured variability via ASV.** The subheadings “Cardiovascular Death” and “Composite Outcomes” are followed by the reports included in the respective sub-analysis. The number of participants in the most variable group are shown in the column “Group Size”. CV Death RR = 1.51; 95% CI 1.19 – 1.92; P = 0.0008; I^2^ = 49.75; P for heterogeneity = 0.1379. Most composite CV outcome RR = 1.62; 95% CI 1.28 – 2.06; P < 0.0001; I^2^ = 33.26%; P for heterogeneity = 0.2209.


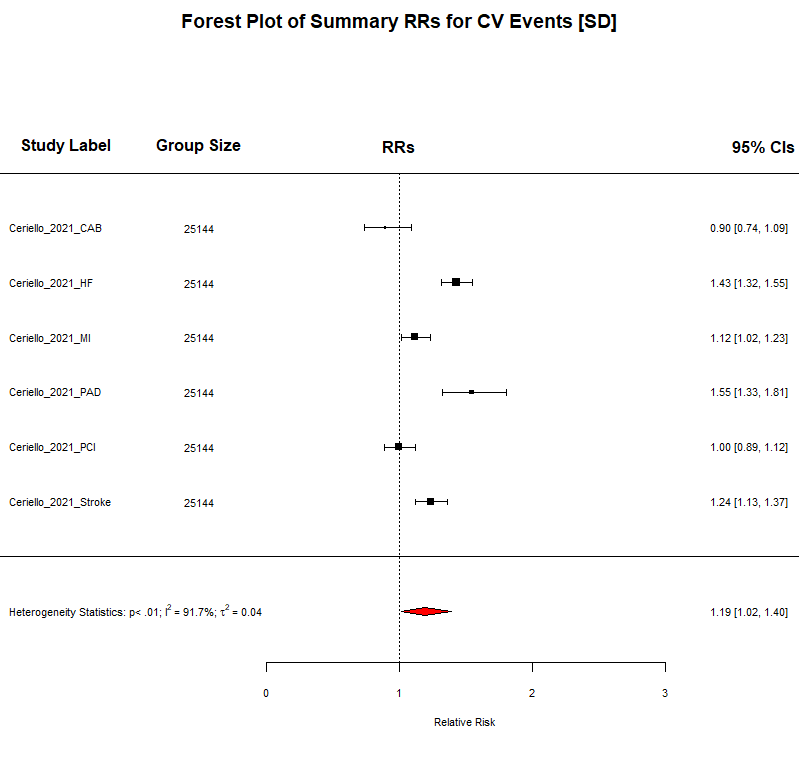


**Figure S5e: Forest plot showing the summative risk of any cardiovascular event associated with being in the top quantile of body weight variability in studies that measured variability via SD.** RR = 1.19; 95% CI 1.02 – 1.40; P = 0.0317; Significant Heterogeneity (I^2^ = 91.73%; P < 0.0001).


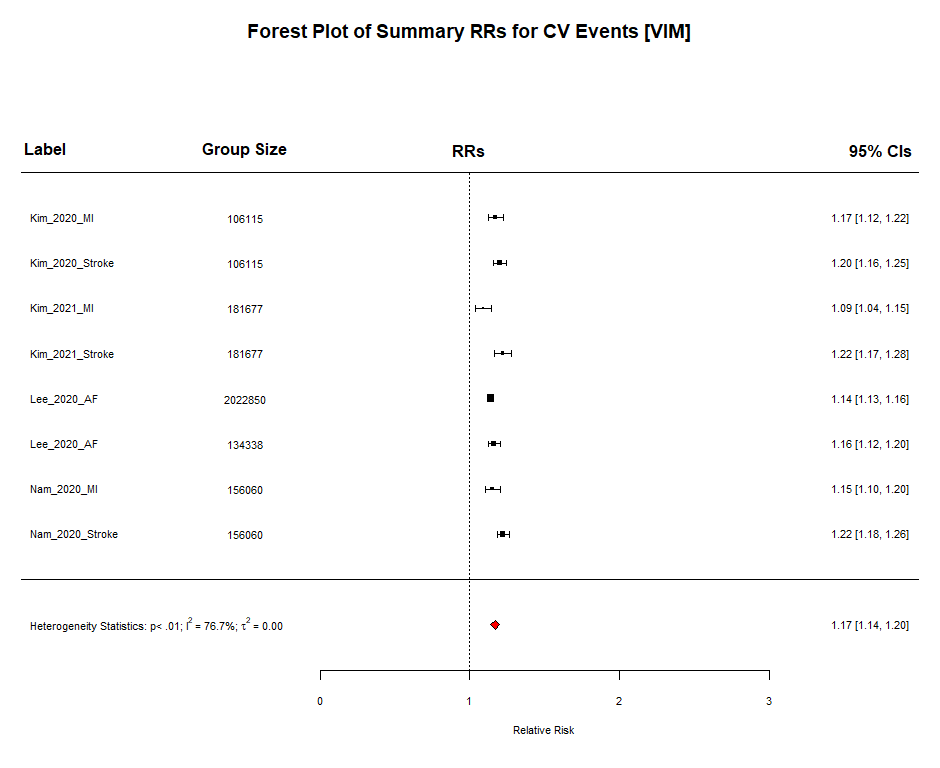


**Figure S5f: Forest plot showing the summative risk of any cardiovascular event associated with being in the top quantile of body weight variability in studies that measured variability via VIM.** RR = 1.17; 95% CI 1.14 – 1.20; P < 0.0001; Significant Heterogeneity (I^2^ = 76.69%; P = 0.0001).


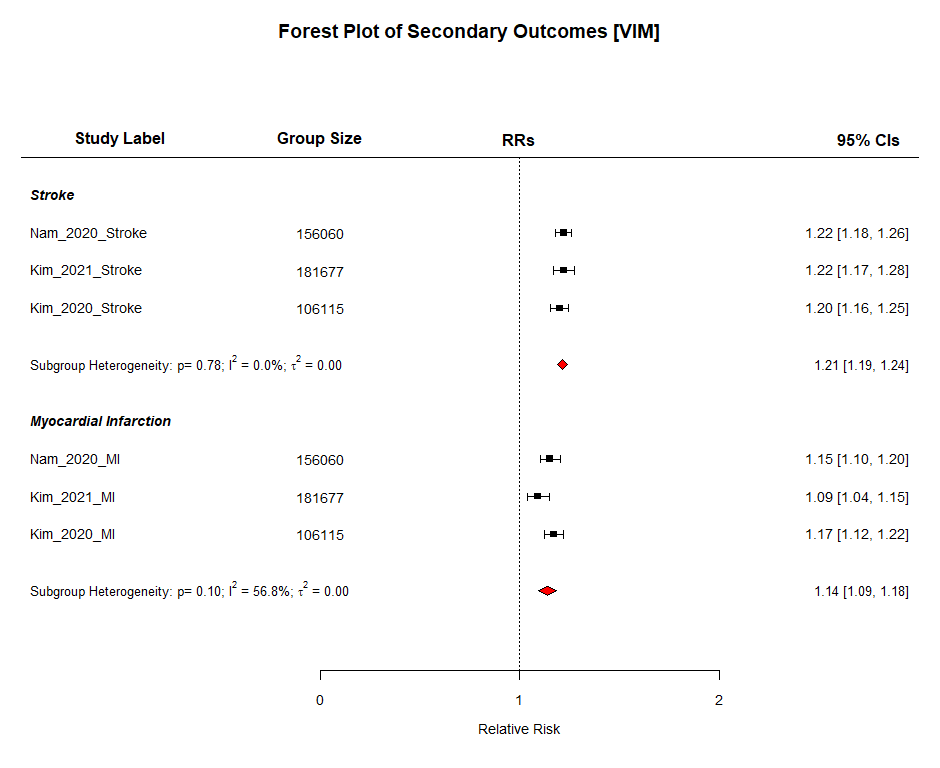


**Figure S5g: A compound forest plot showing the summative risk of the secondary outcomes associated with being in the top quantile of body weight variability in studies that measured variability via VIM.** The subheadings “Stroke” and “Myocardial Infarction” are followed by the reports included in the respective sub-analysis. The number of participants in the most variable group are shown in the column “Group Size”. MI RR = 1.14; 95% CI 1.09 – 1.18; P < 0.0001; I^2^ = 56.84%; P for heterogeneity = 0.1018. Stroke RR = 1.21; 95% CI 1.19 – 1.24; P < 0.0001; I^2^ = 0.00%; P for heterogeneity = 0.7787.


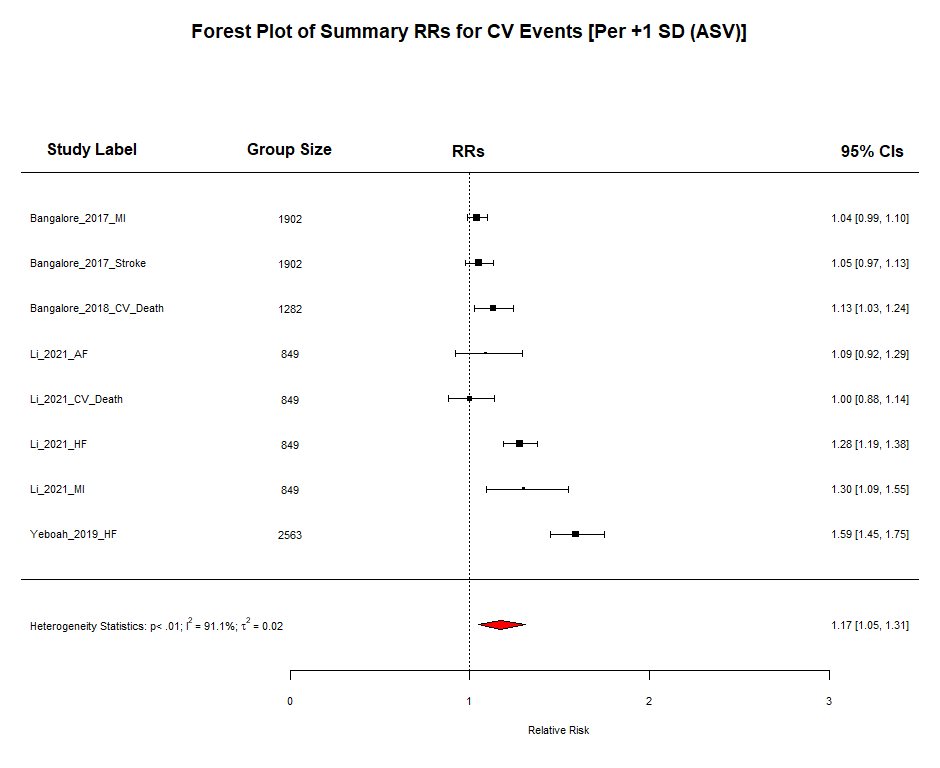


**Figure S5h: Forest plot showing the summative risk of any cardiovascular event associated per +1 SD increase in body weight variability in studies that measured variability via ASV.** RR = 1.17; 95% CI 1.05 – 1.31; P = 0.005; Significant Heterogeneity (I^2^ = 91.13%; P < 0.0001).


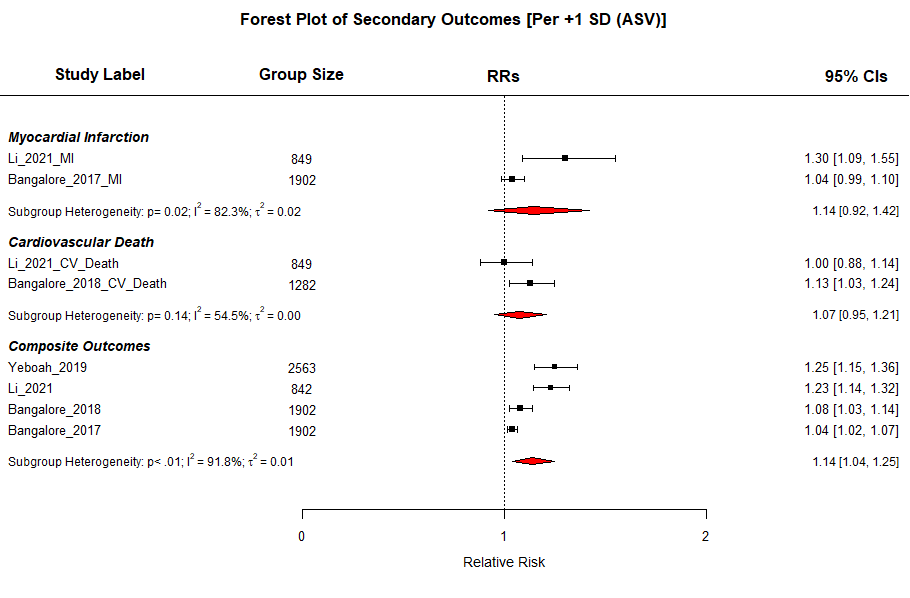


**Figure S5i: A compound forest plot showing the summative risk of the secondary outcomes associated with being in the top quantile of body weight variability in studies that measured variability via VIM.** The subheadings “Myocardial Infarction”, “Cardiovascular Death”, and “Composite Outcomes” are followed by the reports included in the respective sub-analysis. The number of participants in the most variable group are shown in the column “Group Size”. CV Death RR = 1.07; 95% CI 0.95 – 1.21; P = 0.2551; I^2^ = 54.48%; P for heterogeneity = 0.1383. MI RR = 1.14; 95% CI 0.92 – 1.42; P = 0.2234; I^2^ = 82.32%; P for heterogeneity = 0.0174. Most composite CV outcome RR = 1.14; 95% CI 1.04 – 1.25; P = 0.0047; I^2^ = 91.77%; P for heterogeneity < 0.0001.

# Figure S6: Results of Quantile Stratification


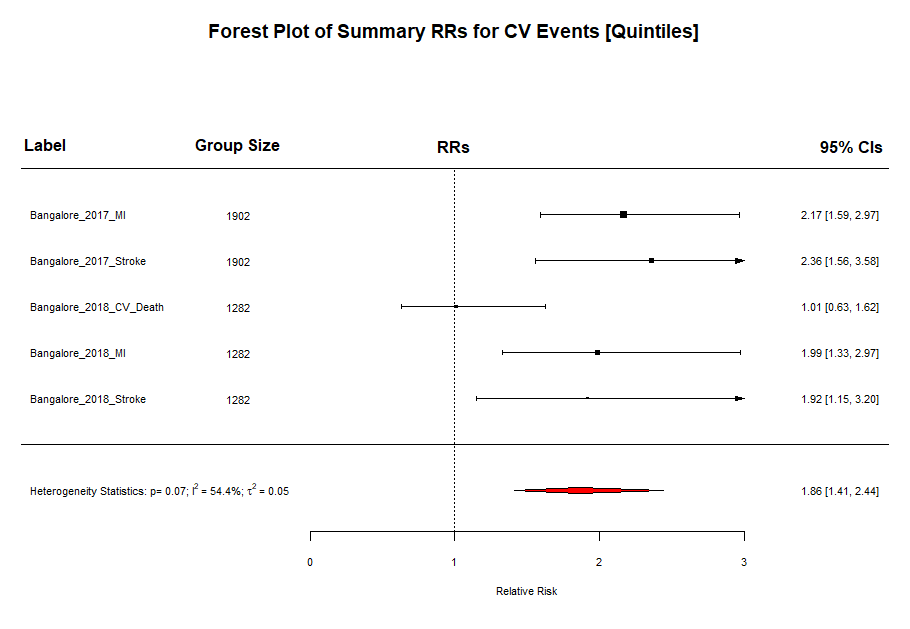


**Figure S6a: Forest plot showing the summative risk of any cardiovascular event associated with being in the top quintile of body weight variability compared to the lowest quintile in studies that compared quintiles of variability.** RR = 1.86; 95% CI 1.41 – 2.44; P < 0.001; Insignificant Heterogeneity (I^2^ = 54.40%; P = 0.0725).


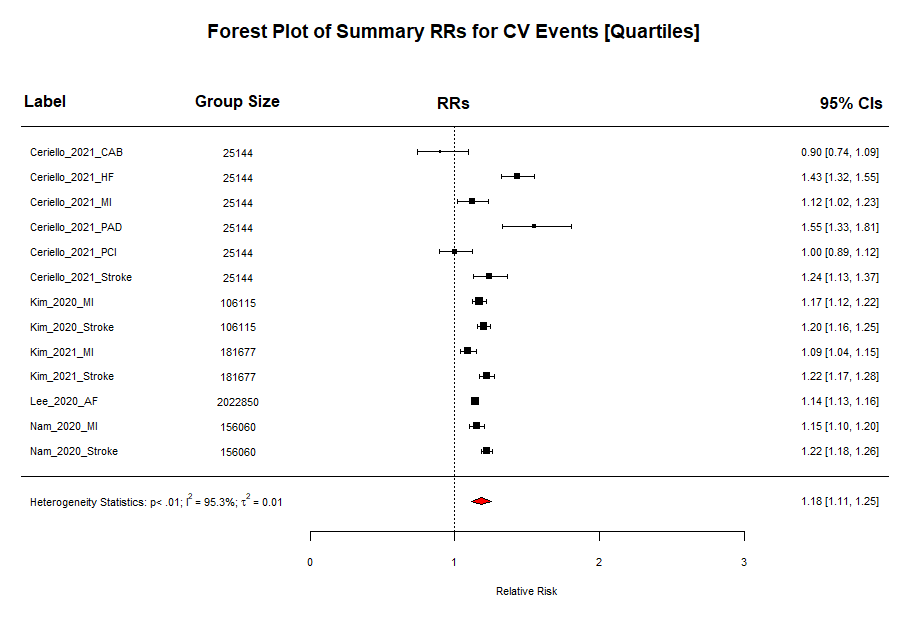


**Figure S6b: Forest plot showing the summative risk of any cardiovascular event associated with being in the top quartile of body weight variability compared to the lowest quartile in studies that compared quartiles of variability.** RR = 1.18; 95% CI 1.11 – 1.25; P < 0.0001; Significant Heterogeneity (I^2^ = 95.30%; P < 0.0001).


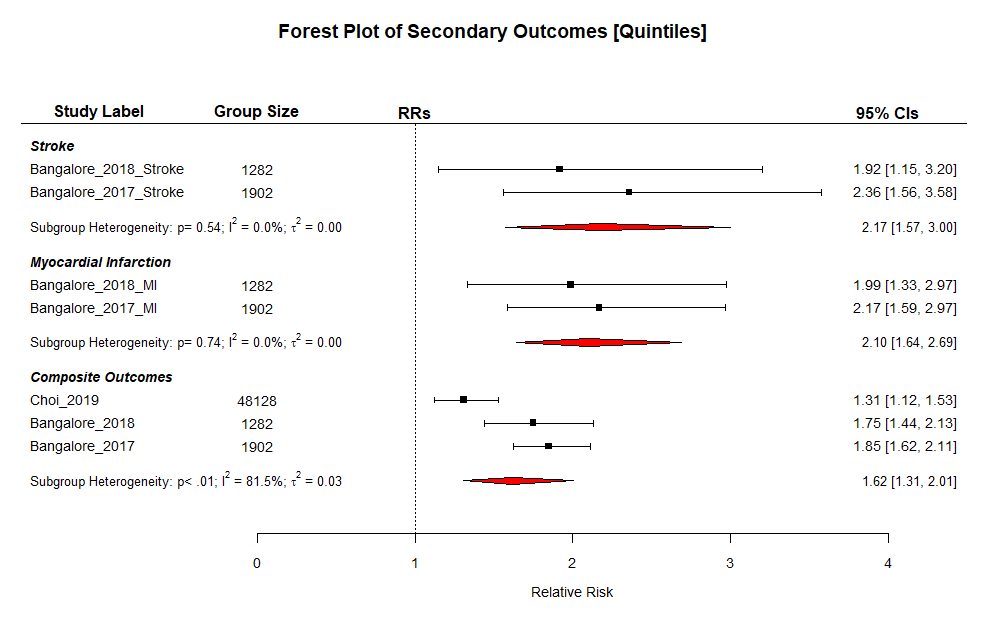


**Figure S6c: A compound forest plot showing the summative risk of the secondary outcomes associated with being in the top quintile of body weight variability compared to the lowest quintile in studies that compared quintiles of variability.** The subheadings “Stroke”, ”Myocardial Infarction”, and “Composite Outcomes” are followed by the reports included in the respective sub-analysis. The number of participants in the most variable group are shown in the column “Group Size”. MI RR = 2.10; 95% CI 1.64 – 2.69; P < 0.0001; I^2^ = 0.00%; P for heterogeneity = 0.7387. Stroke RR = 2.17; 95% CI 1.57 – 3.00; P < 0.0001; I^2^ = 0.00%; P for heterogeneity = 0.5394. Most composite CV outcome RR = 1.62; 95% CI 1.31 – 2.01; P < 0.0001; I^2^ = 81.49%; P for heterogeneity = 0.0031.


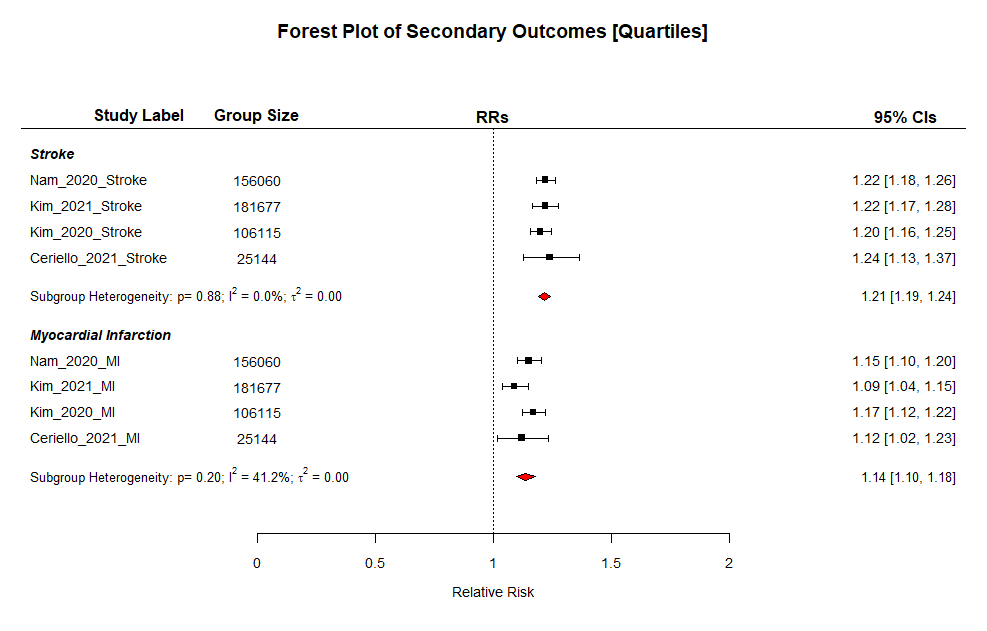


**Figure S6d: A compound forest plot showing the summative risk of the secondary outcomes associated with being in the top quartile of body weight variability compared to the lowest quartile in studies that compared quartiles of variability.** The subheadings “Stroke” and ”Myocardial Infarction” are followed by the reports included in the respective sub-analysis. The number of participants in the most variable group are shown in the column “Group Size”. MI RR = 1.14; 95% CI 1.10 – 1.18; P < 0.0001; I^2^ = 0.00%; P for heterogeneity = 0.5892. Stroke RR = 1.21; 95% CI 1.19 – 1.24; P < 0.0001; I^2^ = 41.24%; P for heterogeneity = 0.1951.

# Figure S7: Results of Previous Cardiovascular Disease Stratification


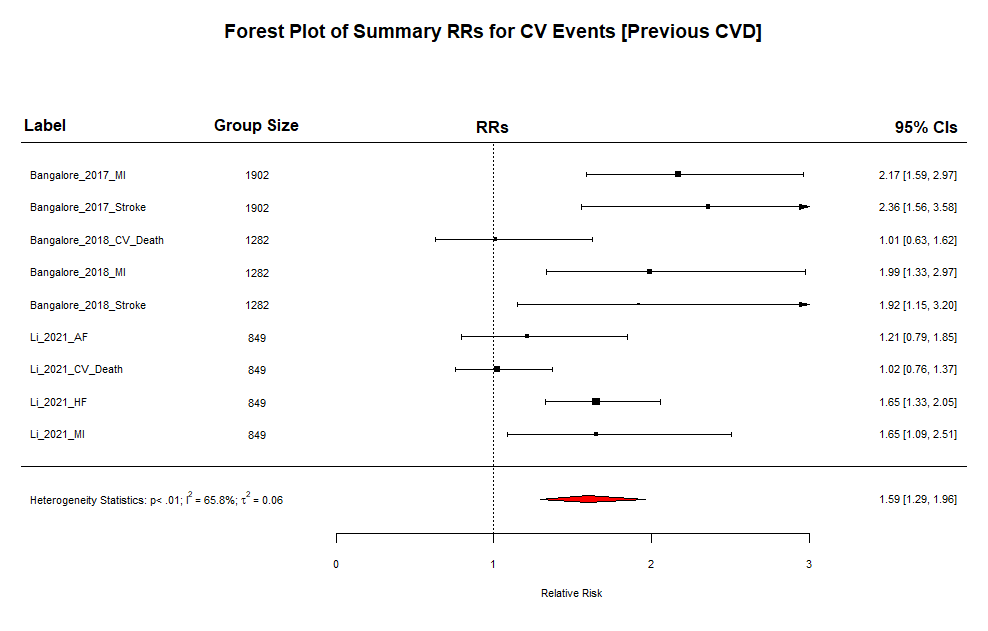


**Figure S7a: Forest plot showing the summative risk of any cardiovascular event associated with being in the top quantile of body weight variability in studies that investigated populations with known prior cardiovascular disease.** RR = 1.59; 95% CI 1.29 – 1.96; P < 0.0001; Significant Heterogeneity (I^2^ = 65.75%; P = 0.0035).


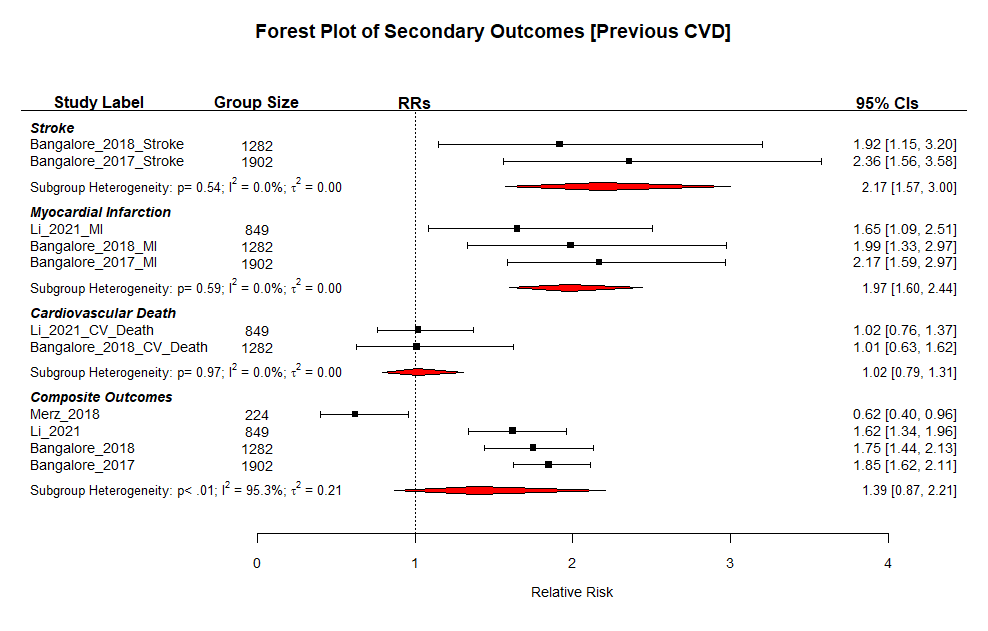


**Figure S7b: A compound forest plot showing the summative risk of the secondary outcomes associated with being in the top quartile of body weight variability in studies associated with being in the top quantile of body weight variability in studies that investigated populations with known prior cardiovascular disease.** The subheadings “Stroke”, ”Myocardial Infarction”, “Cardiovascular Death”, and “Composite Outcomes” are followed by the reports included in the respective sub-analysis. The number of participants in the most variable group are shown in the column “Group Size”. CV Death RR = 1.02; 95% CI 0.79 – 1.31; P = 0.8943; I^2^ = 0.00%; P for heterogeneity = 0.9725. MI RR = 1.97; 95% CI 1.60 – 2.44; P < 0.0001; I^2^ = 0.00%; P for heterogeneity = 0.5892. Stroke RR = 2.17; 95% CI 1.57 – 3.00; P < 0.0001; I^2^ = 0.00%; P for heterogeneity = 0.5394. Most composite CV outcome RR = 1.39; 95% CI 0.87 – 2.21; P < 0.0001; I^2^ = 95.27%; P for heterogeneity < 0.0001.


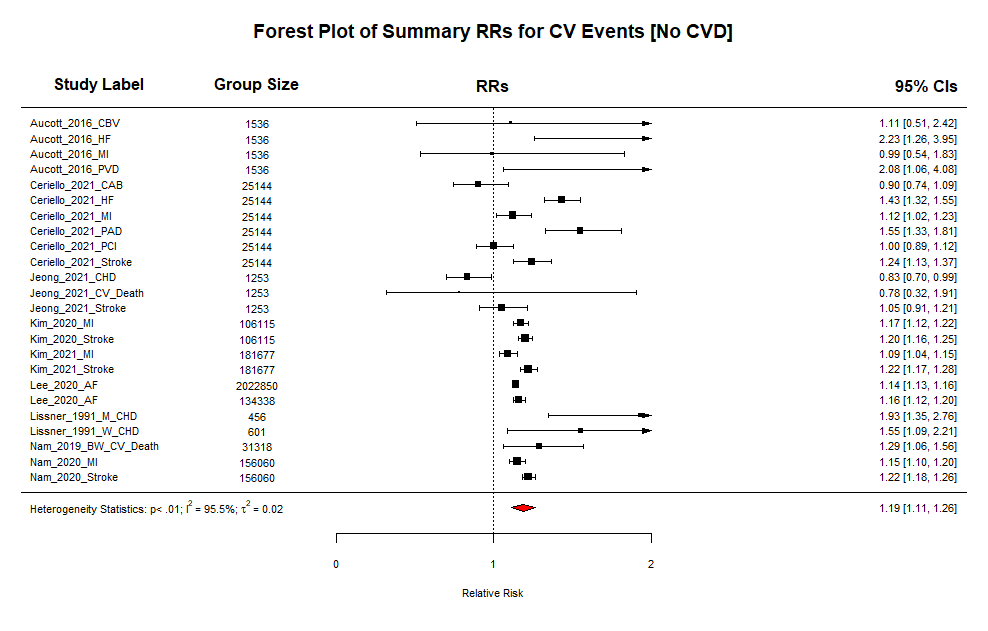


**Figure S7c: Forest plot showing the summative risk of any cardiovascular event associated with being in the top quantile of body weight variability in studies that investigated populations with no known prior cardiovascular disease.** RR = 1.19; 95% CI 1.11 – 1.26; P < 0.0001; Significant Heterogeneity (I^2^ = 95.49%; P < 0.0001).


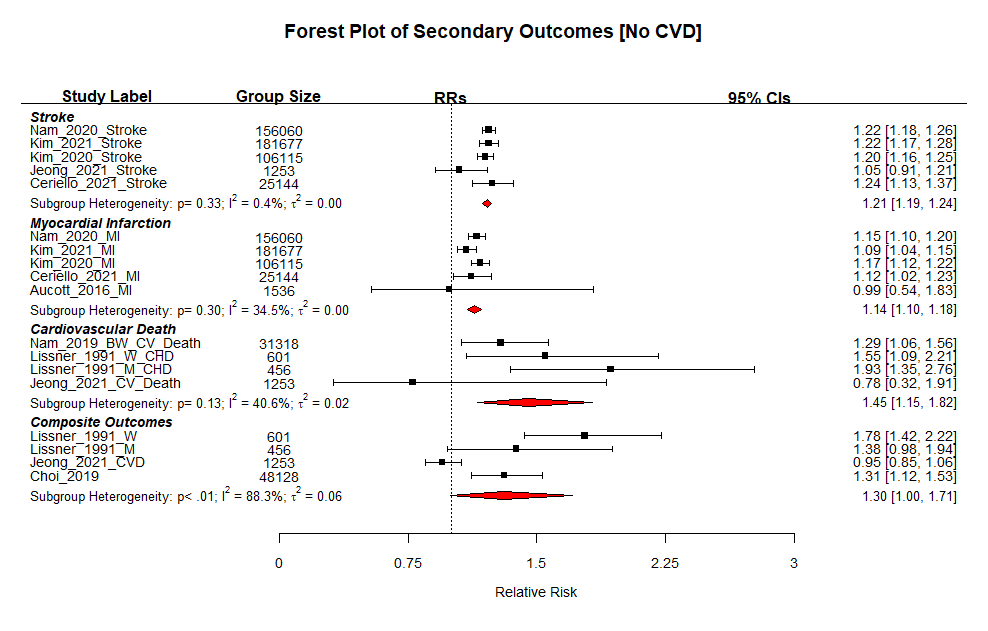


**Figure S7d: A compound forest plot showing the summative risk of the secondary outcomes associated with being in the top quartile of body weight variability in studies associated with being in the top quantile of body weight variability in studies that investigated populations with no known prior cardiovascular disease.** The subheadings “Stroke”, ”Myocardial Infarction”, “Cardiovascular Death”, and “Composite Outcomes” are followed by the reports included in the respective sub-analysis. The number of participants in the most variable group are shown in the column “Group Size”. CV Death RR = 1.45; 95% CI 1.15 – 1.82; P = 0.0016; I^2^ = 40.58%; P for heterogeneity = 0.1267. MI RR = 1.14; 95% CI 1.10 – 1.18; P < 0.0001; I^2^ = 34.50%; P for heterogeneity = 0.2974. Stroke RR = 1.21; 95% CI 1.19 – 1.24; P < 0.0001; I^2^ = 0.43%; P for heterogeneity = 0.3294. Most composite CV outcome RR = 1.30; 95% CI 1.00 – 1.71; P = 0.0531; I^2^ = 88.34%; P for heterogeneity < 0.0001.

Figure S8: Results of adjustment for change in BMI or average BMI stratification
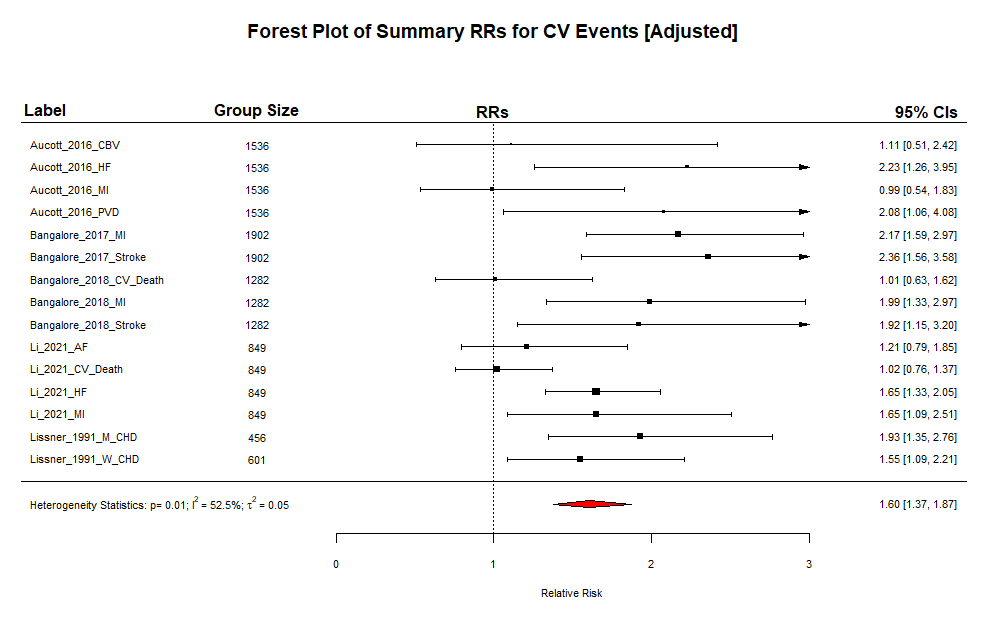


**Figure S8a: Forest plot showing the summative risk of any cardiovascular event associated with being in the top quantile of body weight variability in studies that adjusted for change in BMI or average BMI.** RR = 1.60; 95% CI 1.37 – 1.87; P < 0.0001; Significant Heterogeneity (I^2^ = 52.50%; P = 0.0104).


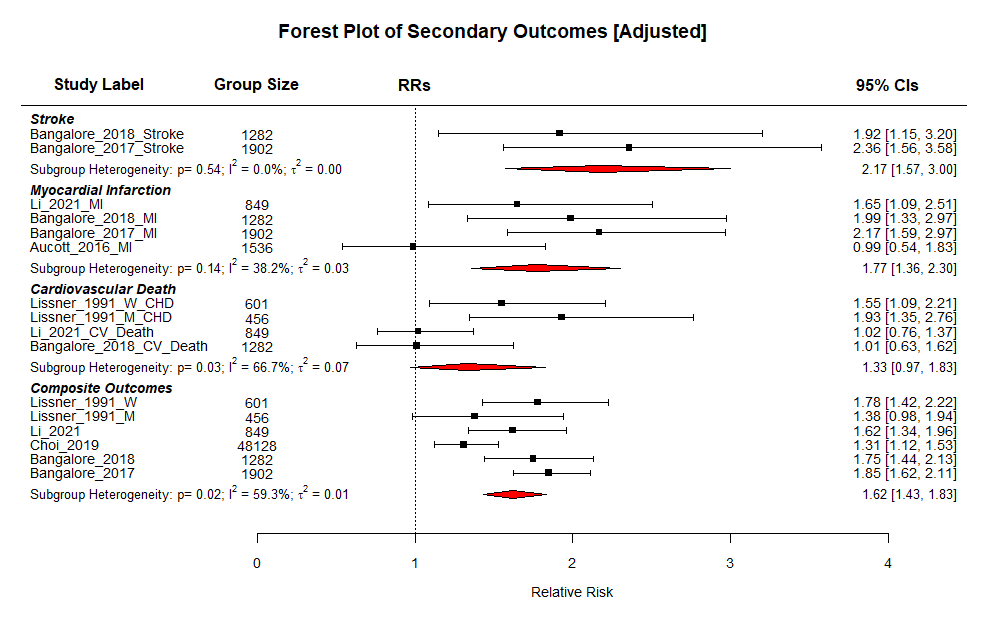


**Figure S8b: A compound forest plot showing the summative risk of the secondary outcomes associated with being in the top quartile of body weight variability in studies that adjusted for change in BMI or average BMI.** The subheadings “Stroke”, ”Myocardial Infarction”, “Cardiovascular Death”, and “Composite Outcomes” are followed by the reports included in the respective sub-analysis. The number of participants in the most variable group are shown in the column “Group Size”. CV Death RR = 1.33; 95% CI 0.97 – 1.83; P = 0.0741; I^2^ = 66.71%; P for heterogeneity = 0.0266. MI RR = 1.77; 95% CI 1.36 – 2.30; P < 0.0001; I^2^ = 38.25%; P for heterogeneity = 0.1445. Stroke RR = 2.17; 95% CI 1.57 – 3.00; P < 0.0001; I^2^ = 0.00%; P for heterogeneity = 0.5394. Most composite CV outcome RR = 1.62; 95% CI 1.43 – 1.83; P < 0.0001; I^2^ = 59.32%; P for heterogeneity = 0.0225.


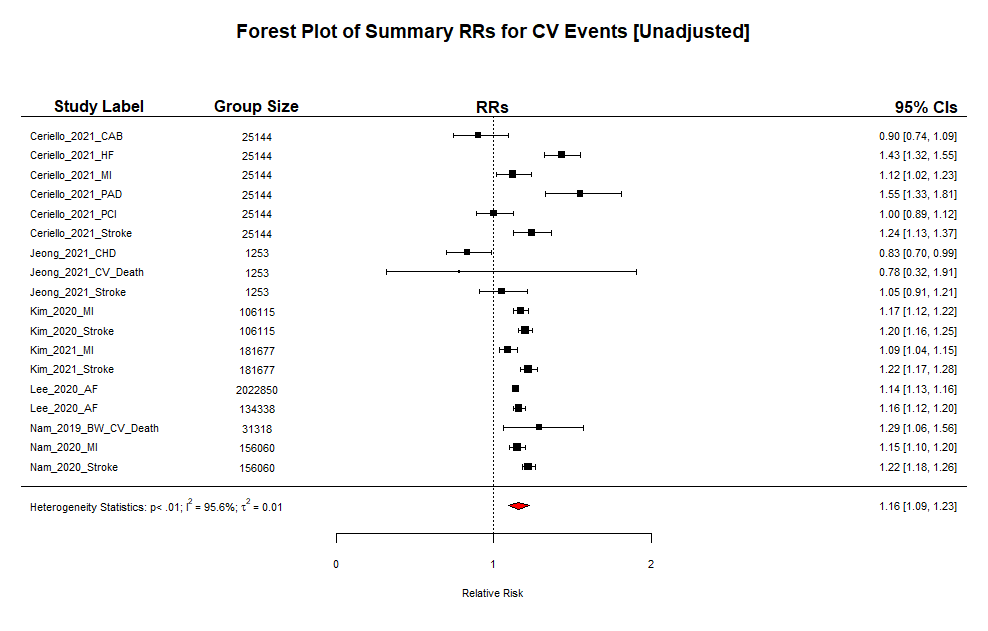


**Figure S8c: Forest plot showing the summative risk of any cardiovascular event associated with being in the top quantile of body weight variability in studies that did not adjust for change in BMI or average BMI.** RR = 1.16; 95% CI 1.09 – 1.23; P < 0.0001; Significant Heterogeneity (I^2^ = 95.63%; P < 0.0001).


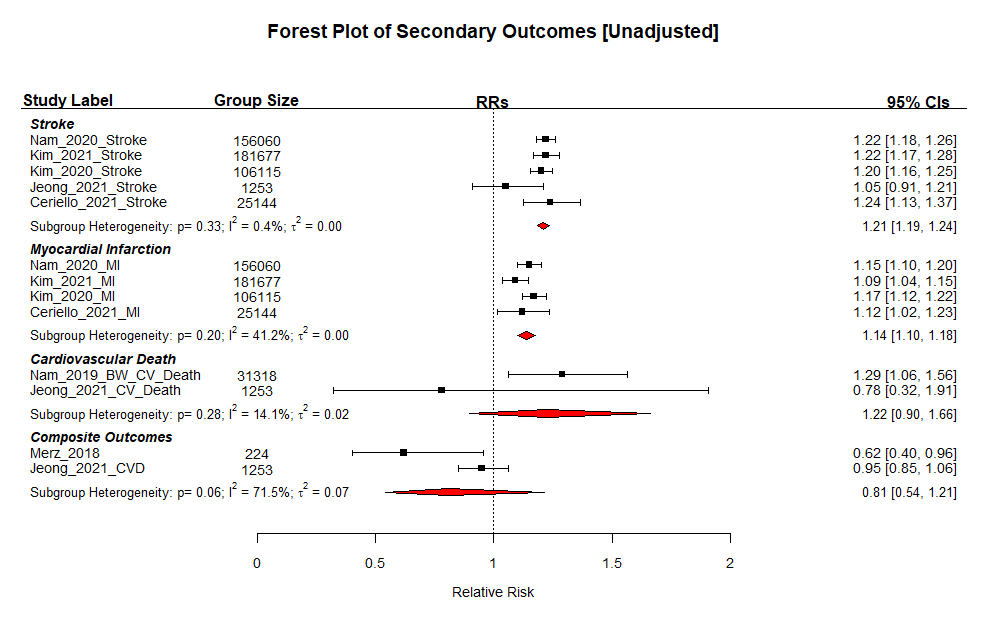


**Figure S8d: A compound forest plot showing the summative risk of the secondary outcomes associated with being in the top quartile of body weight variability in studies that did not adjust for change in BMI or average BMI.** The subheadings “Stroke”, ”Myocardial Infarction”, “Cardiovascular Death”, and “Composite Outcomes” are followed by the reports included in the respective sub-analysis. The number of participants in the most variable group are shown in the column “Group Size”. CV Death RR = 1.22; 95% CI 0.90 – 1.66; P = 0.2022; I^2^ = 14.10%; P for heterogeneity = 0.2806. MI RR = 1.14; 95% CI 1.10 – 1.18; P < 0.0001; I^2^ = 41.24%; P for heterogeneity = 0.1951. Stroke RR = 1.21; 95% CI 1.19 – 1.24; P < 0.0001; I^2^ = 0.43%; P for heterogeneity = 0.3294. Most composite CV outcome RR = 0.81; 95% CI 0.54 – 1.21; P = 0.3064; I^2^ = 71.52%; P for heterogeneity = 0.0610.

Figure S9: Results of Univariate Meta-Regression by Age
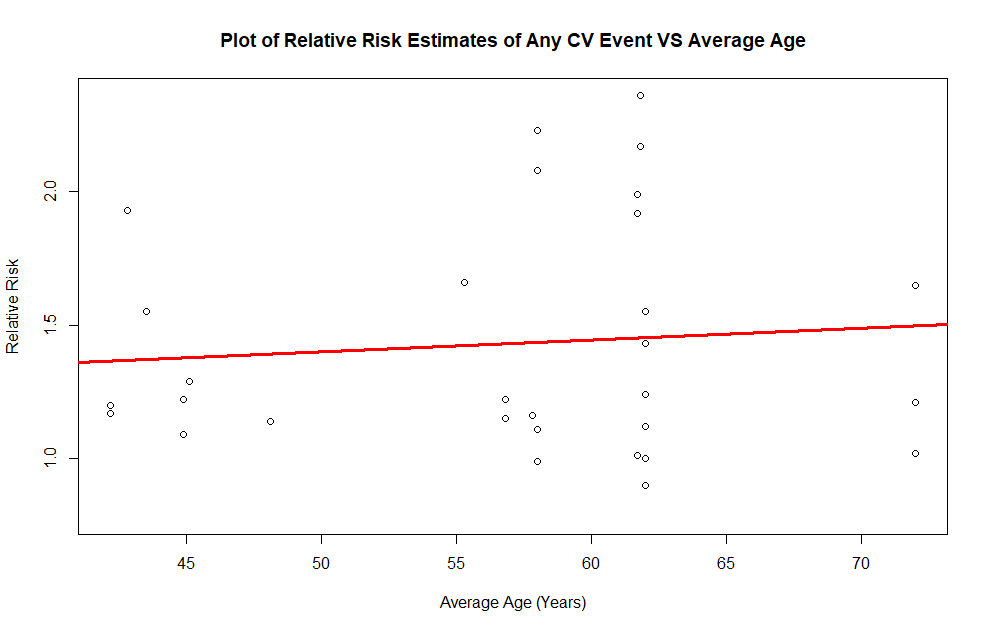


**Figure S9a: A scatter plot showing the univariate meta-regression analysis on how the average age reported by studies affects the relative risk of any cardiovascular event.** Age coefficient = 0.0081; P = 0.0345; R^2^ = 0.028.


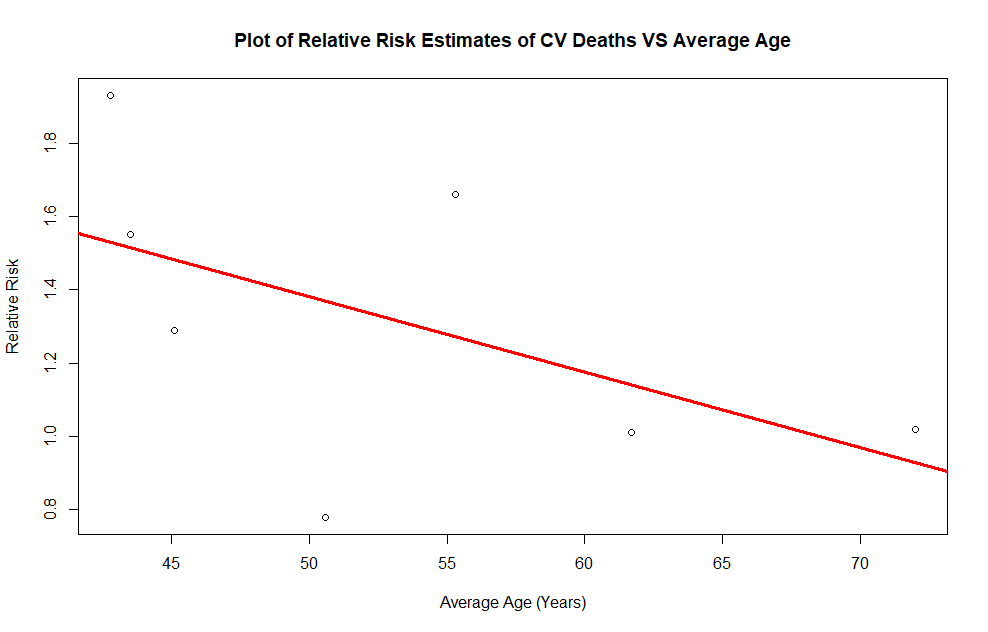


**Figure S9b: A scatter plot showing the univariate meta-regression analysis on how the average age reported by studies affects the relative risk of cardiovascular death.** Age coefficient = -0.0206; P = 0.2106; R^2^ = 0.292.


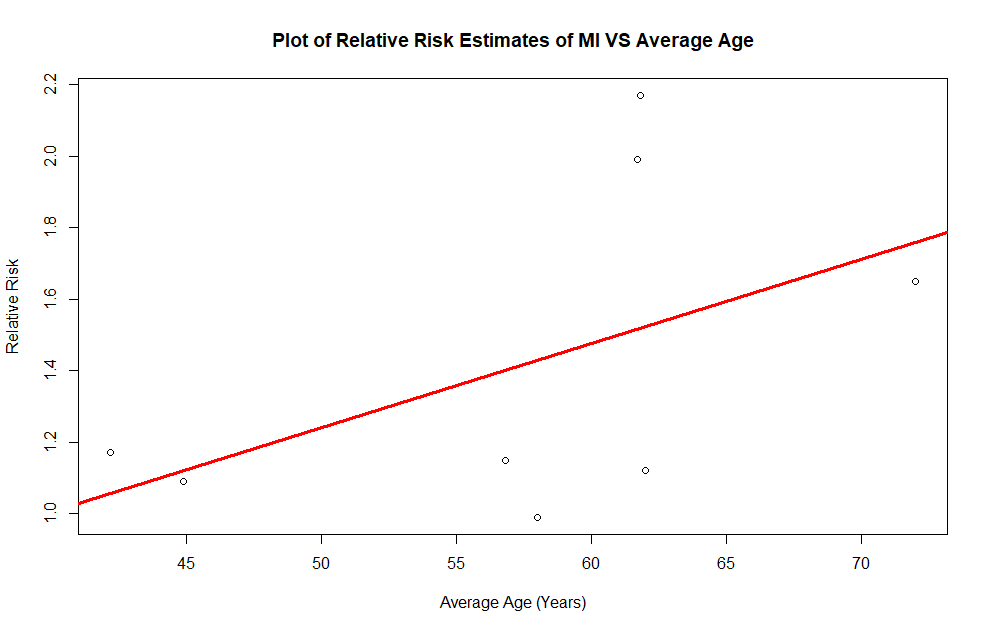


**Figure S9c: A scatter plot showing the univariate meta-regression analysis on how the average age reported by studies affects the relative risk of myocardial infarction.** Age coefficient = 0.0234; P = 0.2057; R^2^ = 0.251.


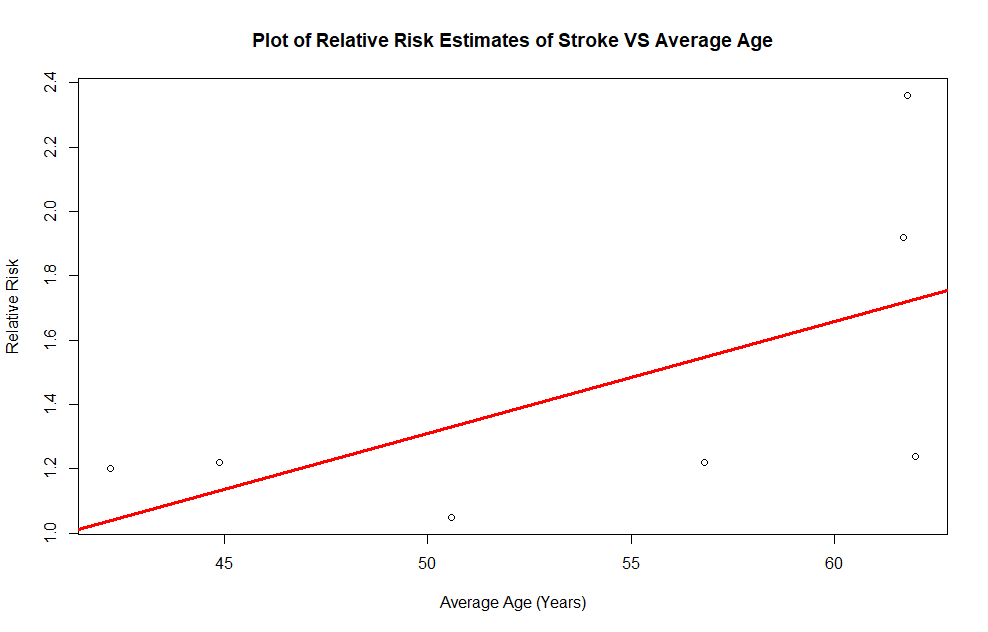


**Figure S9d: A scatter plot showing the univariate meta-regression analysis on how the average age reported by studies affects the relative risk of stroke.** Age coefficient = 0.0348; P = 0.153; R^2^ = 0.362.


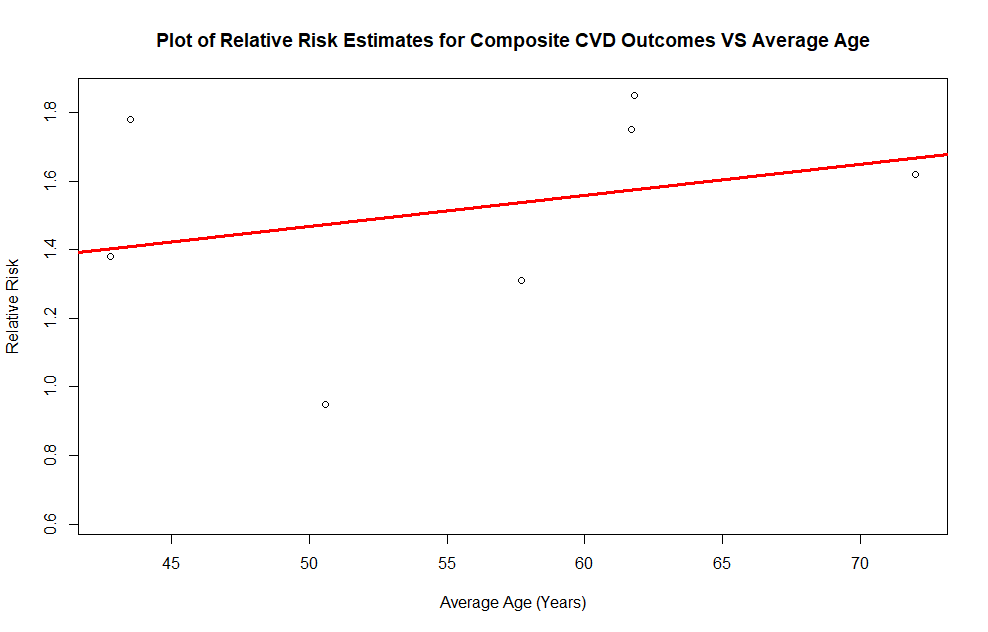


**Figure S9e: A scatter plot showing the univariate meta-regression analysis on how the average age reported by studies affects the relative risk of composite cardiovascular outcomes.** Age coefficient = 0.009; P = 0.5179; R^2^ = 0.088.

# Figure S10: Egger’s Regression and Funnel Plots


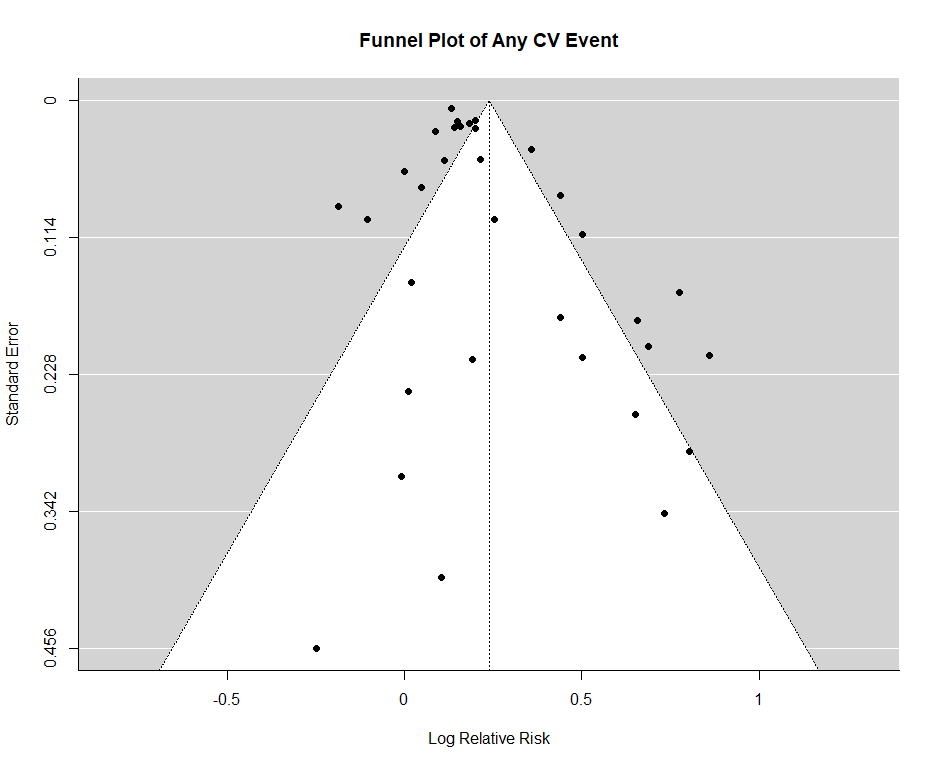


**Figure S10a: A funnel plot of the studies include in the analysis of the RR of any cardiovascular event associated with being in the top quantile of body weight variability.** Egger’s Regression test for funnel plot asymmetry found insignificant asymmetry: z = 1.7567; P = 0.079.


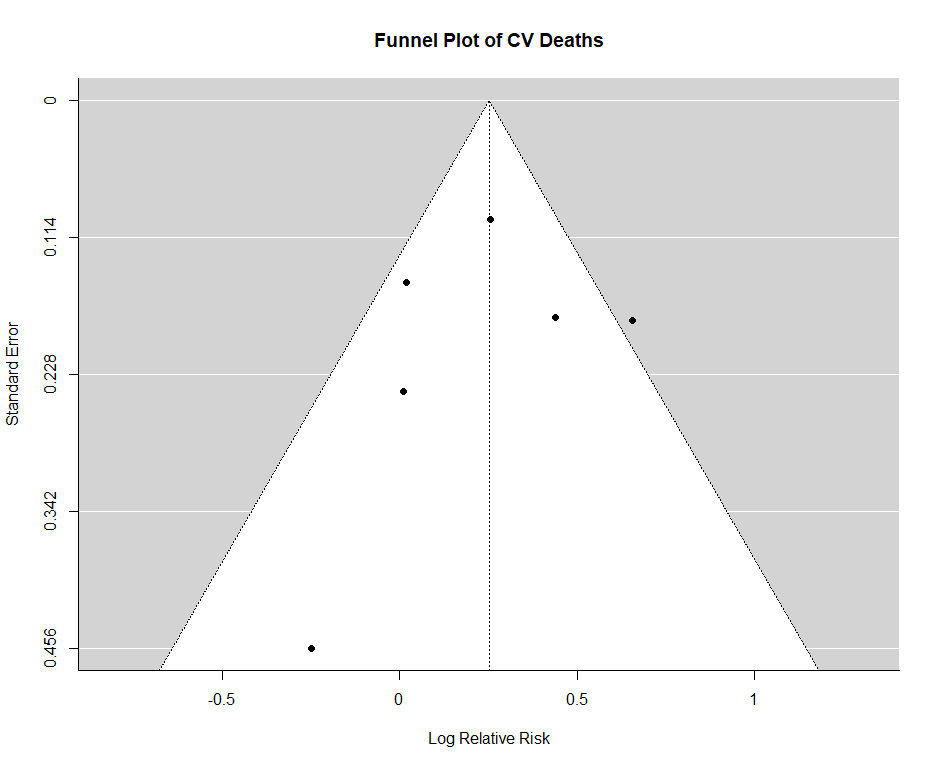


**Figure S10b: A funnel plot of the studies include in the analysis of the RR of cardiovascular death associated with being in the top quantile of body weight variability.** Egger’s Regression test for funnel plot asymmetry found insignificant asymmetry: z = -1.0027; P = 0.316.


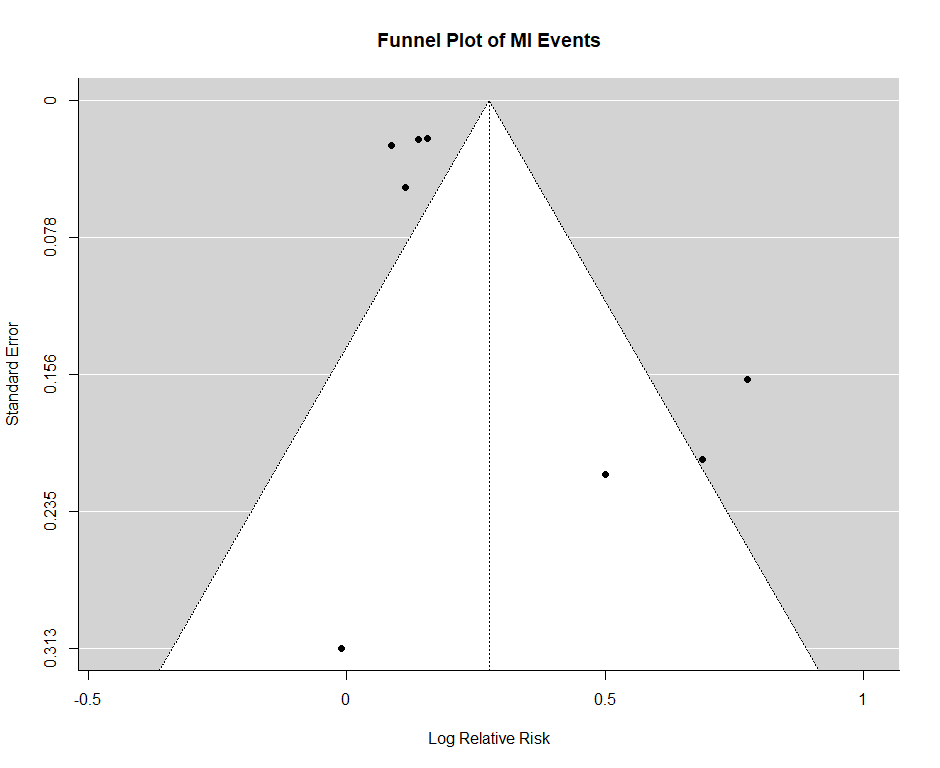


**Figure S10c: A funnel plot of the studies include in the analysis of the RR of myocardial infarction associated with being in the top quantile of body weight variability.** Egger’s Regression test for funnel plot asymmetry found insignificant asymmetry: z = 1.1849; P = 0.236.


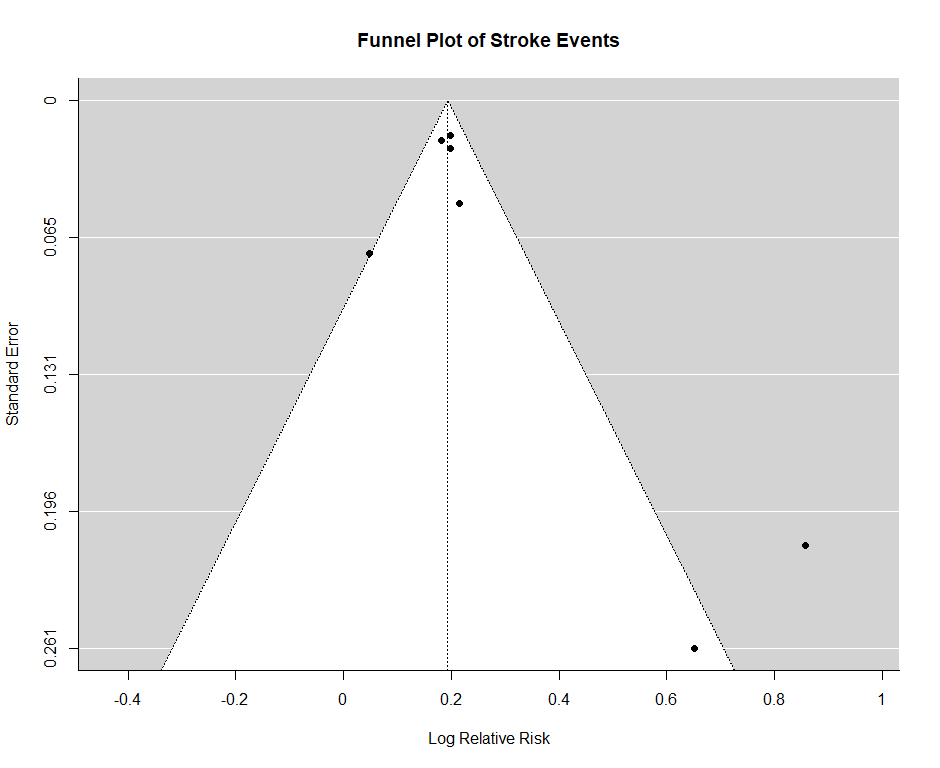


**Figure S10d: A funnel plot of the studies include in the analysis of the RR of stroke associated with being in the top quantile of body weight variability.** Egger’s Regression test for funnel plot asymmetry found significant asymmetry: z = 2.9287; P = 0.0034.


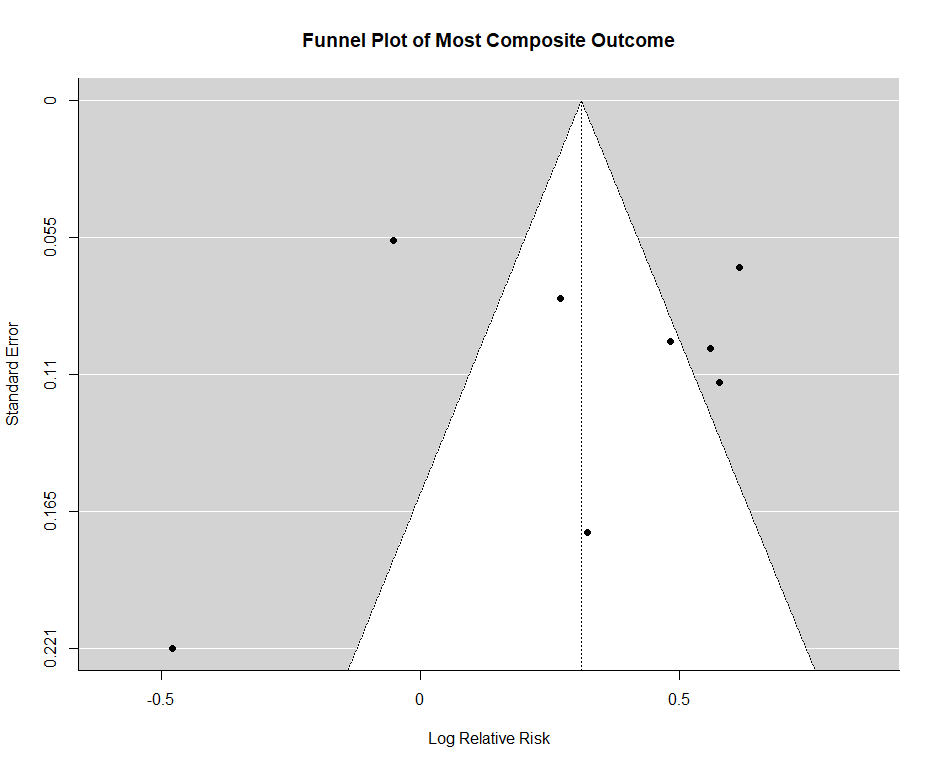


**Figure S10e: A funnel plot of the studies include in the analysis of the RR of the most composite cardiovascular outcome associated with being in the top quantile of body weight variability.** Egger’s Regression test for funnel plot asymmetry found insignificant asymmetry: z = -1.7294; P = 0.0837.


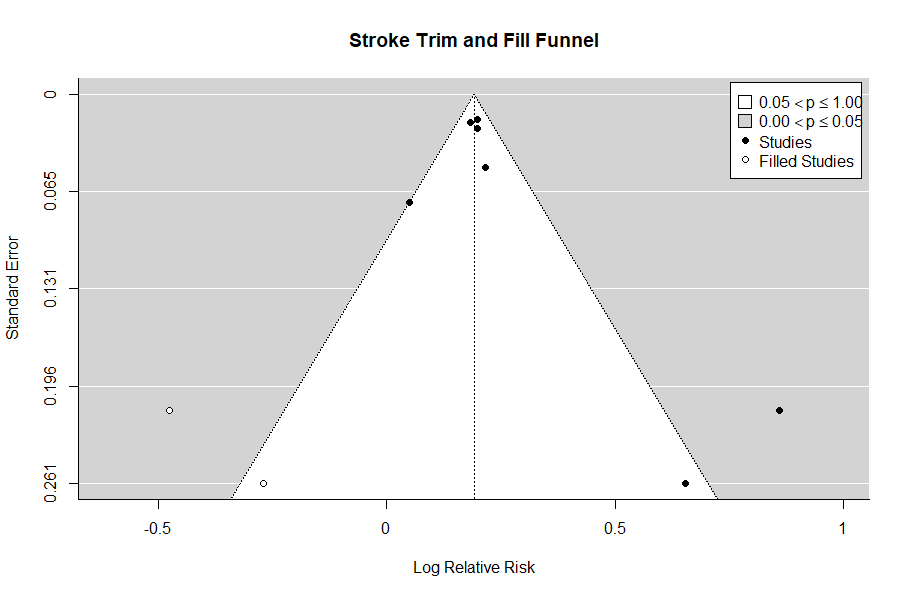


**Figure S10f: A Duval and Tweedie trim-and-fill funnel plot of the studies include in the analysis of the RR of Stroke associated with being in the top quantile of body weight variability.** Estimated number of missing studies on the left side: 2 (SE = 1.8916); model-predicted RR = 1.21; 95% CI 1.19 – 1.24; P < 0.0001. Significant heterogeneity (I^2^ = 0.00%; P = 0.0002).

# Figure S11: Results of Newcastle-Ottawa Bias Analysis


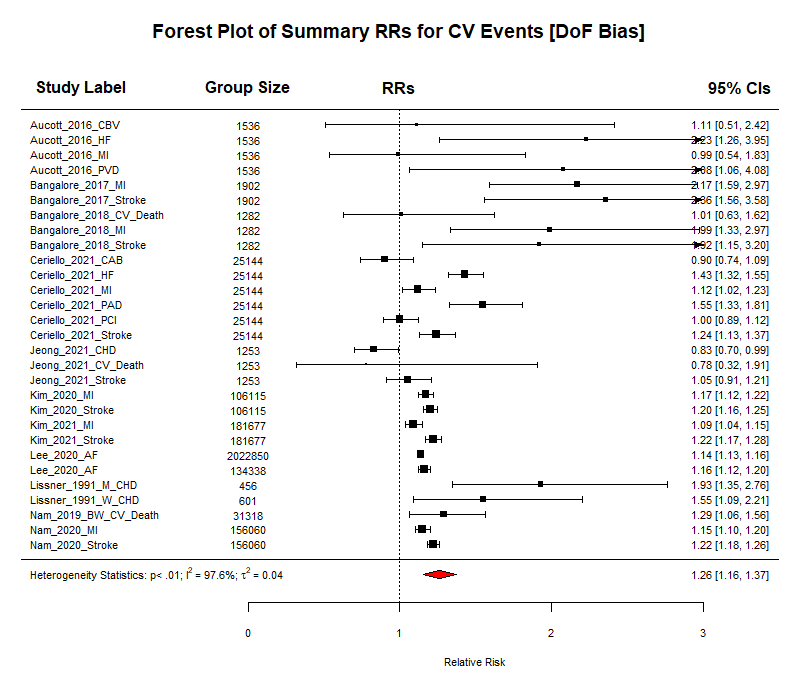


**Figure S11a: Forest plot showing the summative risk of any cardiovascular event associated with being in the top quantile of body weight variability. Only includes studies that scored ≥ 7 on the Newcastle-Ottawa Scale for Quality Assessment of Cohort Studies.** RR = 1.26; 95% CIs 1.16 – 1.37; P < 0.0001; Significant Heterogeneity (I^2^ = 97.64%; P < 0.0001).


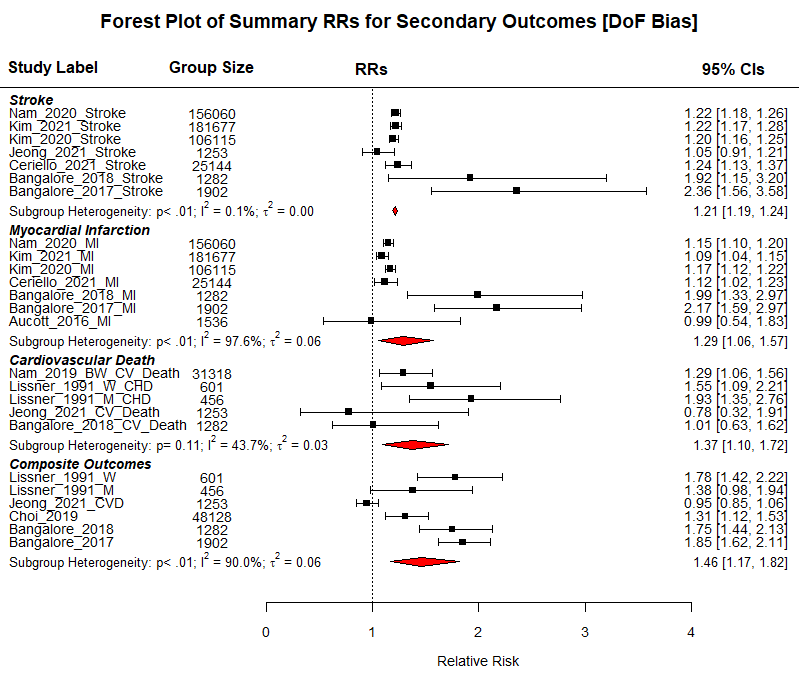


**Figure S11b: A compound forest plot showing the summative risk of the secondary outcomes associated with being in the top quantile of body weight variability. Only includes studies that scored ≥ 7 on the Newcastle-Ottawa Scale for Quality Assessment of Cohort Studies.** The subheadings “Stroke”, ”Myocardial Infarction”, “Cardiovascular Death”, and “Composite Outcomes” are followed by the reports included in the respective sub-analysis. The number of participants in the most variable group are shown in the column “Group Size”. CV Death RR = 1.37; 95% CI 1.10 – 1.72; P = 0.0054; I^2^ = 43.67%; P for heterogeneity = 0.1143. MI RR = 1.29; 95% CI 1.06 – 1.57; P = 0.0122; I^2^ = 97.64%; P for heterogeneity < 0.0001. Stroke RR = 1.21; 95% CI 1.19 – 1.24; P < 0.0001; I^2^ = 0.06%; P for heterogeneity = 0.0073. Most composite CV outcome RR = 1.46; 95% CI 1.17 – 1.82; P = 0.0007; I^2^ = 90.02%; P for heterogeneity < 0.0001.


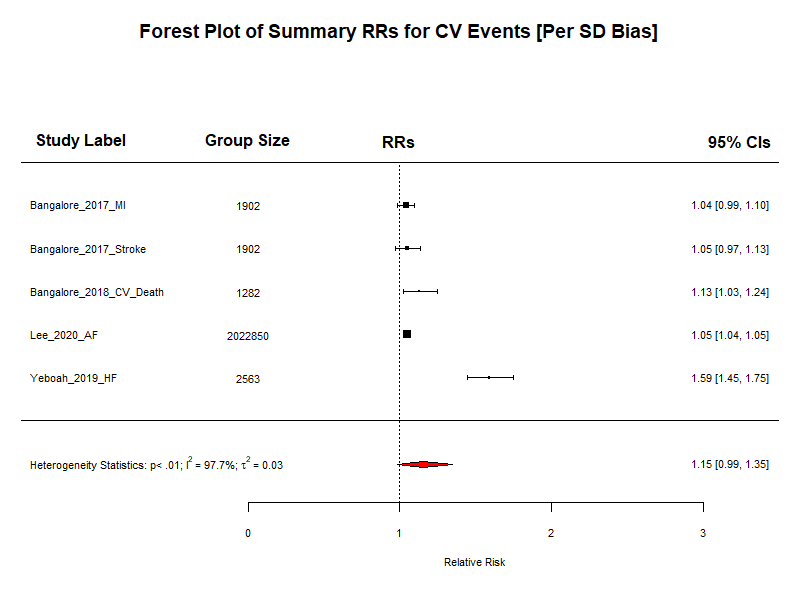


**Figure S11c: Forest plot showing the summative risk of any cardiovascular event associated per +1 SD increase in body weight variability. Only includes studies that scored ≥ 7 on the Newcastle-Ottawa Scale for Quality Assessment of Cohort Studies.** RR = 1.15; 95% CIs 0.99 – 1.35; P = 0.0763; Significant Heterogeneity (I^2^ = 97.74%; P < 0.0001).


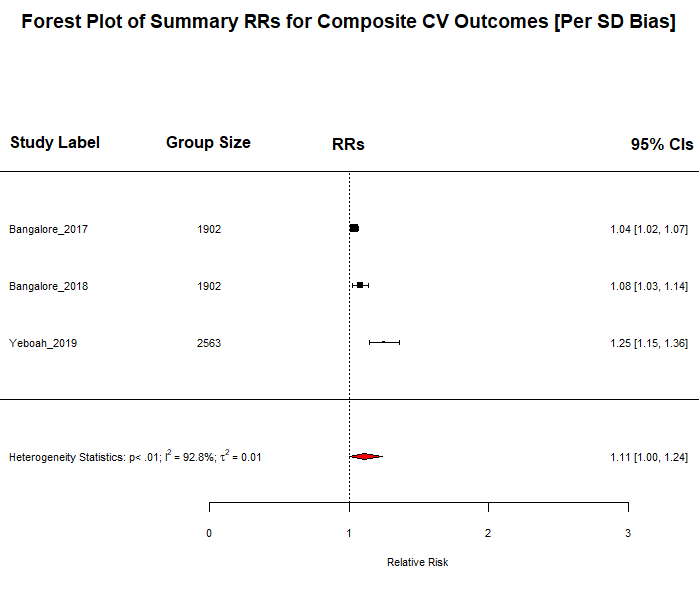


**Figure S11d: Forest plot showing the summative risk of the most composite outcome recorded by included studies associated per +1 SD increase in body weight variability. Only includes studies that scored ≥ 7 on the Newcastle-Ottawa Scale for Quality Assessment of Cohort Studies.** RR = 1.11; 95% CIs 1.00 – 1.24; P = 0.0459; Significant Heterogeneity (I^2^ = 92.80%; P = 0.0001).


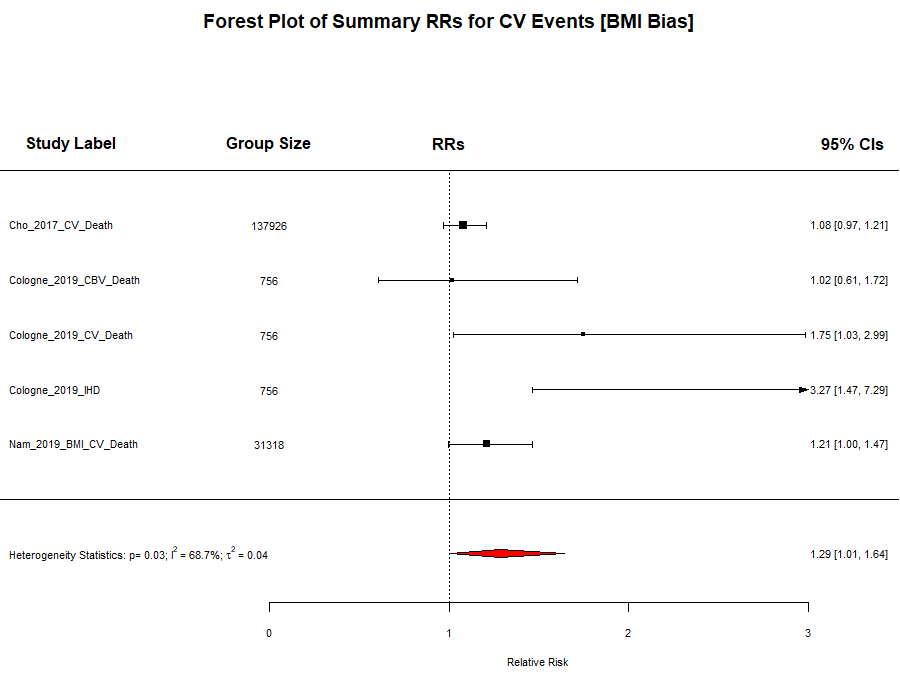


**Figure S11h: Forest plot showing the summative risk of cardiovascular death associated with being in the top quantile of BMI variability. Only includes studies that scored ≥ 7 on the Newcastle-Ottawa Scale for Quality Assessment of Cohort Studies.** RR = 1.29; 95% CIs 1.01 – 1.64; P = 0.0446; Significant Heterogeneity (I^2^ = 68.65%; P = 0.0319).

# Appendix 4: MOOSE Checklist

| **Item No** | **Recommendation** | **Reported on Page No** |
| --- | --- | --- |
| Reporting of background should include | | |
| 1 | Problem definition | 3 |
| 2 | Hypothesis statement | 3 |
| 3 | Description of study outcome(s) | 5 |
| 4 | Type of exposure or intervention used | 4-5 |
| 5 | Type of study designs used | 4 |
| 6 | Study population | 4 |
| Reporting of search strategy should include | | |
| 7 | Qualifications of searchers (eg, librarians and investigators) | Not Reported |
| 8 | Search strategy, including time period included in the synthesis and key words | 4, Appendix 1 |
| 9 | Effort to include all available studies, including contact with authors | 4 |
| 10 | Databases and registries searched | 4 |
| 11 | Search software used, name and version, including special features used (eg, explosion) | 6 |
| 12 | Use of hand searching (eg, reference lists of obtained articles) | None |
| 13 | List of citations located and those excluded, including justification | 8 |
| 14 | Method of addressing articles published in languages other than English | 5 |
| 15 | Method of handling abstracts and unpublished studies | None |
| 16 | Description of any contact with authors | Appendix 2 |
| Reporting of methods should include | | |
| 17 | Description of relevance or appropriateness of studies assembled for assessing the hypothesis to be tested | 4 - 5 |
| 18 | Rationale for the selection and coding of data (eg, sound clinical principles or convenience) | 5 |
| 19 | Documentation of how data were classified and coded (eg, multiple raters, blinding and interrater reliability) | 5 |
| 20 | Assessment of confounding (eg, comparability of cases and controls in studies where appropriate) | 7 |
| 21 | Assessment of study quality, including blinding of quality assessors, stratification or regression on possible predictors of study results | 7 |
| 22 | Assessment of heterogeneity | 7 |
| 23 | Description of statistical methods (eg, complete description of fixed or random effects models, justification of whether the chosen models account for predictors of study results, dose-response models, or cumulative meta-analysis) in sufficient detail to be replicated | 6 |
| 24 | Provision of appropriate tables and graphics | 6 |
| Reporting of results should include | | |
| 25 | Graphic summarizing individual study estimates and overall estimate | Figure 2, Figure 3 |
| 26 | Table giving descriptive information for each study included | Table S2 |
| 27 | Results of sensitivity testing (eg, subgroup analysis) | 12 - 14 |
| 28 | Indication of statistical uncertainty of findings | Yes |

| **Item No** | **Recommendation** | **Reported on Page No** |
| --- | --- | --- |
| Reporting of discussion should include | | |
| 29 | Quantitative assessment of bias (eg, publication bias) | 13 – 14, Figures S6 & S7 |
| 30 | Justification for exclusion (eg, exclusion of non-English language citations) | Figure 1 |
| 31 | Assessment of quality of included studies | Table S3 |
| Reporting of conclusions should include | | |
| 32 | Consideration of alternative explanations for observed results | 17 |
| 33 | Generalization of the conclusions (ie, appropriate for the data presented and within the domain of the literature review) | 17 |
| 34 | Guidelines for future research | 17 |
| 35 | Disclosure of funding source | 17 |

*From*: Stroup DF, Berlin JA, Morton SC, et al, for the Meta-analysis Of Observational Studies in Epidemiology (MOOSE) Group. Meta-analysis of Observational Studies in Epidemiology. A Proposal for Reporting. *JAMA*. 2000;283(15):2008-2012. doi: 10.1001/jama.283.15.2008.

# Table S4: Newcastle – Ottawa Scale Quality Assessment Results

| **Study Label** | **Representativeness of the exposed cohort** | **Selection of the non-exposed cohort** | **Ascertainment of Exposure** | **Demonstration that outcome of interest was not present at the start of the study** | **Comparability of cohorts on the basis of the design or analysis** | **Assessment of Outcome** | **Was follow-up long enough for outcomes to occur** | **Adequacy of follow up of cohorts** | **Total Score** |
| --- | --- | --- | --- | --- | --- | --- | --- | --- | --- |
| **Aucott et al., 2016** | b | a | b | b | b | c | a | b | **8** |
| **Bangalore et al., 2017** | b | a | a | a | b | b | a | d | **7** |
| **Bangalore et al., 2018** | b | a | a | a | b | b | a | d | **7** |
| **Ceriello et al., 2021** | a | a | a | a | a + b | b | a | d | **8** |
| **Cho et al., 2017** | a | a | a | a | a + b | b | a | b | **9** |
| **Choi et al., 2019** | b | a | a | b | a + b | b | a | b | **8** |
| **Cologne et al, 2019** | b | a | a | a | a + b | b | a | d | **8** |
| **Diaz et al., 2005** | b | a | c | b | a + b | b | a | d | **6** |
| **Dyer et al., 2000** | b | a | d | a | b | b | a | c | **6** |
| **Jeong et al., 2021** | b | a | a | a | a + b | b | a | b | **9** |
| **Kim et al., 2020** | b | a | a | a | a + b | b | a | b | **9** |
| **Kim et al., 2021** | b | a | a | a | a + b | b | a | c | **8** |
| **Lee et al., 2020** | b | a | a | a | a + b | a | a | b | **9** |
| **Lee et al., 2020** | b | a | a | a | a + b | a | a | d | **8** |
| **Li et al., 2021** | b | a | a | b | b | b | a | d | **6** |
| **Lissner et al., 1991** | b | a | a | a | b | a | a | d | **7** |
| **Merz et al., 2018** | b | s | b | b | b | c | a | b | **6** |
| **Nam et al., 2019** | b | a | a | b | a + b | b | a | b | **8** |
| **Nam et al., 2020** | b | a | a | a | a + b | a | a | c | **8** |
| **Sponholtz et al., 2019** | b | a | b | a | b | d | a | d | **6** |
| **Wannamethee et al., 2002** | b | a | c | b | a + b | d | a | c | **5** |
| **Yeboah et al., 2019** | b | a | a | a | a + b | a | a | a | **9** |
| **Youk et al., 2020** | b | a | a | b | b | b | a | d | **6** |

**Table S3: Results of quality assessment of the individual included studies using the Newcastle-Ottawa Scale**

# Appendix 5: Newcastle-Ottawa Quality Assessment Scale

**COHORT STUDIES**

**Selection**

1) Representativeness of the exposed cohort

a) truly representative of the average ____body-weight/BMI _____ (describe) in the community **🟑**

b) somewhat representative of the average _____body-weight/BMI________ in the community **🟑**

c) selected group of users eg nurses, volunteers

d) no description of the derivation of the cohort

2) Selection of the non exposed cohort

a) drawn from the same community as the exposed cohort **🟑**

b) drawn from a different source

c) no description of the derivation of the non exposed cohort

3) Ascertainment of exposure

a) secure record (eg surgical records) **🟑**

b) structured interview **🟑**

c) written self report

d) no description

4) Demonstration that outcome of interest was not present at start of study

a) yes **🟑**

b) no

**Comparability**

1) Comparability of cohorts on the basis of the design or analysis

a) study controls for __baseline bodyweight/BMI__ (select the most important factor) **🟑**

b) study controls for any additional factor **🟑** (This criteria could be modified to indicate specific control for a second important factor.)

**Outcome**

1) Assessment of outcome

a) independent blind assessment **🟑**

b) record linkage **🟑**

c) self report

d) no description

2) Was follow-up long enough for outcomes to occur

a) yes (select an adequate follow up period for outcome of interest [3 years]) **🟑**

b) no

3) Adequacy of follow up of cohorts

a) complete follow up - all subjects accounted for **🟑**

b) subjects lost to follow up unlikely to introduce bias - small number lost - > _80_ % (select an adequate %) follow up, or description provided of those lost) **🟑**

c) follow up rate < _80_% (select an adequate %) and no description of those lost

d) no statement

*From:* The Newcastle-Ottawa Scale (NOS) for assessing the quality of nonrandomised studies in meta-analyses [Internet]. 2021. Available from: http://www.ohri.ca/programs/clinical_epidemiology/oxford.asp.

# References

1. Aucott LS, Philip S, Avenell A, Afolabi E, Sattar N, Wild S. Patterns of weight change after the diagnosis of type 2 diabetes in Scotland and their relationship with glycaemic control, mortality and cardiovascular outcomes: a retrospective cohort study. BMJ Open. 2016;6(7):e010836.

2. Lissner L, Odell PM, D'Agostino RB, Stokes J, 3rd, Kreger BE, Belanger AJ, et al. Variability of body weight and health outcomes in the Framingham population. N Engl J Med. 1991;324(26):1839-44.

3. Nam GE, Cho KH, Han K, Han B, Cho SJ, Roh YK, et al. Impact of body mass index and body weight variabilities on mortality: a nationwide cohort study. Int J Obes (Lond). 2019;43(2):412-23.

4. Bangalore S, Fayyad R, Laskey R, DeMicco DA, Messerli FH, Waters DD. Body-Weight Fluctuations and Outcomes in Coronary Disease. New England Journal of Medicine. 2017;376(14):1332-40.

5. Bangalore S, Fayyad R, DeMicco DA, Colhoun HM, Waters DD. Body Weight Variability and Cardiovascular Outcomes in Patients With Type 2 Diabetes Mellitus. Circ Cardiovasc Qual Outcomes. 2018;11(11):e004724.

6. Choi D, Choi S, Park SM. Impact of weight variability on mortality among Korean men and women: a population based study. Scientific Reports. 2019;9(1):9543.

7. Li Y, Yu Y, Wu Y, Liang W, Dong B, Xue R, et al. Association of Body-Weight Fluctuation With Outcomes in Heart Failure With Preserved Ejection Fraction. Front Cardiovasc Med. 2021;8:689591-.

8. Yeboah P, Hsu FC, Bertoni AG, Yeboah J. Body Mass Index, Change in Weight, Body Weight Variability and Outcomes in Type 2 Diabetes Mellitus (from the ACCORD Trial). Am J Cardiol. 2019;123(4):576-81.

9. Youk TM, Kang MJ, Song SO, Park E-C. Effects of BMI and LDL-cholesterol change pattern on cardiovascular disease in normal adults and diabetics. BMJ Open Diabetes Research &amp; Care. 2020;8(2):e001340.

10. Cologne J, Takahashi I, French B, Nanri A, Misumi M, Sadakane A, et al. Association of Weight Fluctuation With Mortality in Japanese Adults. JAMA Netw Open. 2019;2(3):e190731.

11. Dyer AR, Stamler J, Greenland P. Associations of weight change and weight variability with cardiovascular and all-cause mortality in the Chicago Western Electric Company Study. Am J Epidemiol. 2000;152(4):324-33.

12. Ceriello A, Lucisano G, Prattichizzo F, Eliasson B, Franzén S, Svensson A-M, et al. Variability in body weight and the risk of cardiovascular complications in type 2 diabetes: results from the Swedish National Diabetes Register. Cardiovascular Diabetology. 2021;20(1):173.

13. Kim DH, Nam GE, Han K, Kim YH, Park KY, Hwang HS, et al. Variabilities in Weight and Waist Circumference and Risk of Myocardial Infarction, Stroke, and Mortality: A Nationwide Cohort Study. Endocrinol Metab (Seoul). 2020;35(4):933-42.

14. Kim MN, Han K, Yoo J, Ha Y, Chon YE, Lee JH, et al. Body weight variability and the risk of cardiovascular outcomes in patients with nonalcoholic fatty liver disease. Scientific Reports. 2021;11(1):9154.

15. Lee H-J, Choi E-K, Han K-D, Kim DH, Lee E, Lee S-R, et al. High variability in bodyweight is associated with an increased risk of atrial fibrillation in patients with type 2 diabetes mellitus: a nationwide cohort study. Cardiovascular diabetology. 2020;19(1):78-.

16. Lee HJ, Choi EK, Han KD, Lee E, Moon I, Lee SR, et al. Bodyweight fluctuation is associated with increased risk of incident atrial fibrillation. Heart Rhythm. 2020;17(3):365-71.

17. Nam GE, Kim W, Han K, Lee CW, Kwon Y, Han B, et al. Body Weight Variability and the Risk of Cardiovascular Outcomes and Mortality in Patients With Type 2 Diabetes: A Nationwide Cohort Study. Diabetes Care. 2020;43(9):2234-41.
